# HDAC inhibitors enhance neratinib activity and when combined enhance the actions of an anti-PD-1 immunomodulatory antibody *in vivo*

**DOI:** 10.18632/oncotarget.21660

**Published:** 2017-10-09

**Authors:** Laurence Booth, Jane L. Roberts, Andrew Poklepovic, Francesca Avogadri-Connors, Richard E. Cutler, Alshad S. Lalani, Paul Dent

**Affiliations:** ^1^ Department of Biochemistry and Molecular Biology, Virginia Commonwealth University, Richmond, VA 23298, USA; ^2^ Department of Medicine, Virginia Commonwealth University, Richmond, VA 23298, USA; ^3^ Puma Biotechnology Inc., Los Angeles, CA 90024, USA

**Keywords:** autophagy, HDAC, receptor tyrosine kinase, neratinib

## Abstract

Patients whose NSCLC tumors become afatinib resistant presently have few effective therapeutic options to extend their survival. Afatinib resistant NSCLC cells were sensitive to clinically relevant concentrations of the irreversible pan-HER inhibitor neratinib, but not by the first generation ERBB1/2/4 inhibitor lapatinib. In multiple afatinib resistant NSCLC clones, HDAC inhibitors reduced the expression of ERBB1/3/4, but activated c-SRC, which resulted in higher total levels of ERBB1/3 phosphorylation. Neratinib also rapidly reduced the *expression* of ERBB1/2/3/4, c-MET and of mutant K-/N-RAS; K-RAS co-localized with phosphorylated ATG13 and with cathepsin B in vesicles. Combined exposure of cells to [neratinib + HDAC inhibitors] caused inactivation of mTORC1 and mTORC2, enhanced autophagosome and subsequently autolysosome formation, and caused an additive to greater than additive induction of cell death. Knock down of Beclin1 or ATG5 prevented HDAC inhibitors or neratinib from reducing ERBB1/3/4 and K-/N-RAS expression and reduced [neratinib + HDAC inhibitor] lethality. Neratinib and HDAC inhibitors reduced the expression of multiple HDAC proteins via autophagy that was causal in the reduced expression of PD-L1, PD-L2 and ornithine decarboxylase, and increased expression of Class I MHCA. *In vivo*, neratinib and HDAC inhibitors interacted to suppress the growth of 4T1 mammary tumors, an effect that was enhanced by an anti-PD-1 antibody. Our data support the premises that neratinib lethality can be enhanced by HDAC inhibitors, that neratinib may be a useful therapeutic tool in afatinib resistant NSCLC, and that [neratinib + HDAC inhibitor] exposure facilitates anti-tumor immune responses.

## INTRODUCTION

Over-expression of the epidermal growth factor receptor (EGFR, ERBB1) has for many years been recognized as a biomarker for tumor cell growth, invasion and resistance to chemotherapy [1, and references therein]. Other members of this receptor family, ERBB2, ERBB3 and ERBB4, have also been linked to the oncogenic drug-resistant phenotype [2–4]. As ERBB1 was over-expressed in many tumors, several pharmaceutical companies in the 1990s developed drugs that inhibited ERBB1, e.g. gefitinib, erlotinib [5, 6]. In the clinic, in contrast to the laboratory, tumors that over-expressed ERBB1 did not in general exhibit an exquisite sensitivity to the ERBB1 inhibitory drugs [7]. In part this was because neither gefitinib nor erlotinib inhibited ERBB2, and the use of these drugs could also facilitate compensatory cell-survival-signaling via the formation of ERBB2:ERBB3 complexes, with downstream activation of the cytoprotective PI3K pathway [8, 9]. Other mechanisms for gefitinib / erlotinib failure at tumor control included the activation of other cyto-protective growth factor receptors, e.g. c-MET [10].

The early studies with ERBB1 inhibitors eventually resulted in two new directions of research. One avenue was to synthesize new inhibitors that could block signaling by ERBB1, ERBB2 and ERBB4. A second approach was to understand why some tumor cells exhibited a rapid death response after erlotinib or gefitinib exposure, whilst other tumor cells, expressing equal protein amounts of ERBB1, were relatively insensitive to the drugs. The first clinically approved ERBB1/2/4 inhibitor was lapatinib, and was approved for the treatment of breast cancer in combination with capecitabine [11]. In addition to known activating truncation mutants of ERBB1, primarily found in glioblastoma patients, researchers subsequently identified mutable amino acids in full-length ERBB1 which resulted in the mutant enzyme having a significantly higher basal specific activity [12–15]. Mutant full-length ERBB1 is found in ~10-15% of NSCLC patients, generally in those individuals who have not previously been smokers [16]. Full-length mutant activated ERBB1 has also been detected in glioblastoma, mammary, prostate and head & neck carcinoma patients [17–20].

As the clinical experience with mutant active ERBB1 in NSCLC developed, it was noted that patients who successfully received erlotinib/gefitinib mono-therapy would develop drug resistance ~6-18 months after the initiation of treatment. A major component of the drug-resistance mechanism was attributed to the evolution of a second mutation in the ERBB1 catalytic site which prevented erlotinib/ gefitinib from preventing ATP hydrolysis. Other mechanisms of clinical resistance were found to include up-regulation of other compensatory survival signaling receptors, e.g. c-MET [21].

The present studies were initiated to determine whether the irreversible pan-HER inhibitor neratinib could be utilized, alone or in combination with other agents, to kill multiple clones of afatinib-resistant H1975 cells [1]. We then went on to determine whether neratinib-dependent modulation of immunoregulatory proteins could enhance the actions of checkpoint inhibitory antibodies *in vivo*. Our data demonstrated that neratinib, but not lapatinib, killed the afatinib resistant H1975 clones and that neratinib lethality is enhanced by histone deacetylase (HDAC) inhibitors. And, additionally, that the [neratinib + HDAC inhibitor] combination facilitated checkpoint inhibitor anti-tumor immune responses.

## RESULTS

**Figure 1:**
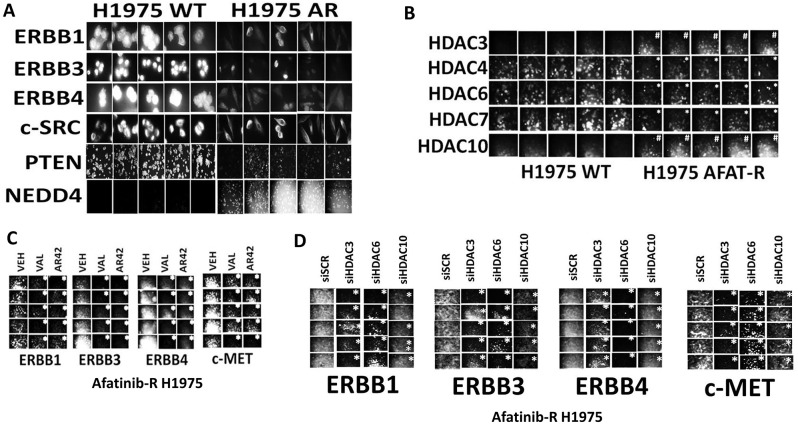
HDAC inhibitors reduce the expression of receptor tyrosine kinases in afatinib resistant NSCLC cells that correlates with inhibition of HDACs1/2/3, HDAC6 and HDAC10 **(A)** Wild type parental H1975 clones and afatinib resistant clones were plated and 24h later fixed in place. Immunofluorescent staining was performed to detect the expression of ERBB1, ERBB3, ERBB4, c-SRC, PTEN and NEDD4 [1]. **(B)** Wild type parental H1975 clones and afatinib resistant clones were plated and 24h later fixed in place. Immunofluorescent staining was performed to detect the expression of HDAC3, HDAC4, HDAC6, HDAC7 and HDAC10. (n = 3 +/-SEM) ^#^ p < 0.05 greater staining intensity than in wild type parental clones; ^*^ p < 0.05 less staining intensity than in wild type parental clones. **(C)** Afatinib resistant H1975 clones were treated with vehicle control, sodium valproate (250 μM) or AR42 (600 nM) for 6h. Cells were fixed in place and immunofluorescent staining performed to detect the expression of ERBB1, ERBB3, ERBB4 and c-MET (n = 3 +/-SEM) ^*^ p < 0.05 less staining intensity than in vehicle control treated clones. **(D)** Afatinib resistant clones were transfected with a scrambled siRNA control or with siRNA molecules to knock down the expression of HDAC3, HDAC6 or HDAC10. Twenty-four h after transfection, cells were fixed in place. Immunofluorescent staining was performed to detect the expression of ERBB1, ERBB3, ERBB4 and c-MET. (n = 3 +/-SEM) ^*^ p < 0.05 less staining intensity than in vehicle control treated clones.

Our initial studies recapitulated some of the previously published descriptive characterizing analyses of our control parental H1975 clones and our afatinib resistant H1975 clones. Afatinib resistant H1975 clones expressed lower levels of ERBB1, ERBB3, ERBB4 and PTEN compared to parental wild type clones (Figure 1A). Afatinib resistant clones expressed slightly lower levels of c-SRC and significantly greater levels of the E3 ligase NEDD4 than wild type clones. Based on these alterations in protein expression, we next investigated whether the levels of histone deacetylase enzymes that regulate transcription were altered in the resistant clones. Afatinib resistant H1975 clones expressed lower levels of HDAC4, HDAC6 and HDAC7, and elevated levels of HDAC3 and HDAC10 (Figure 1B). Treatment of afatinib resistant clones with the HDAC inhibitors sodium valproate or AR42 for 6h significantly reduced the expression of the receptor tyrosine kinases ERBB1, ERBB3, ERBB4 and c-MET (Figure 1C). Molecular knock down of HDAC3, HDAC6 or HDAC10 in the afatinib resistant clones significantly reduced the expression of the receptor tyrosine kinases ERBB1, ERBB3, ERBB4 and c-MET (Figure 1D). Similar findings with respect to receptor tyrosine kinase expression were made when the expression of HDAC1 and of HDAC2 was knocked down (Supplementary Figure 1).

**Figure 2:**
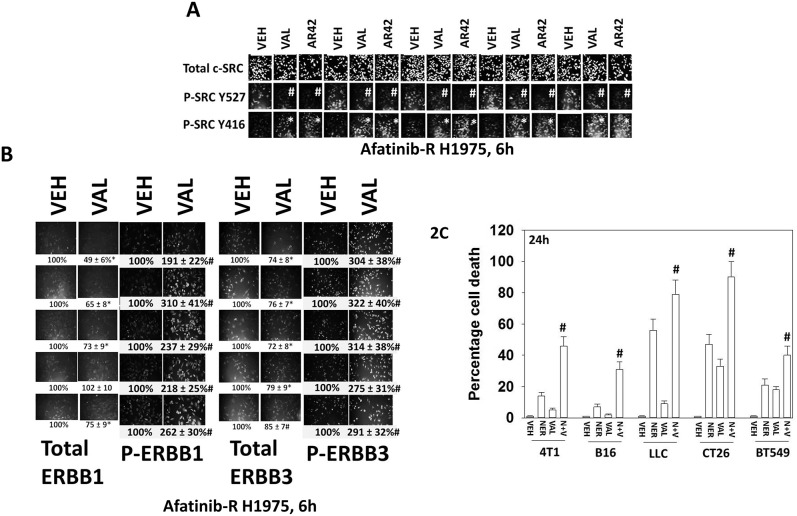
Valproate activates c-SRC/ERBB1/ERBB3 and its anti-tumor activity is enhanced by the ERBB1/2/4 suicide inhibitor neratinib **(A)** Afatinib resistant H1975 clones were treated with vehicle control, sodium valproate (250 μM) or AR42 (600 nM) for 6h. Cells were fixed in place and immunofluorescent staining performed to detect the expression of c-SRC total levels, SRC Y416 phosphorylation, and SRC Y527 phosphorylation (n = 3 +/-SEM) ^*^ p < 0.05 greater staining intensity than in vehicle control treated clones; ^#^ p < 0.05 lower staining intensity than in vehicle control treated clones. **(B)** Afatinib resistant H1975 clones were treated with vehicle control or sodium valproate (250 μM) for 6h. Cells were fixed in place and immunofluorescent staining performed to detect the expression of ERBB1 and ERBB3, and the phosphorylation of ERBB1 (Y1068/Y1173) and of ERBB3 (Y1248). (n = 3 +/-SEM) ^#^ p < 0.05 greater staining intensity than that in vehicle control treated clones; ^*^ p < 0.05 lower staining intensity than in vehicle control treated clones. **(C)** Tumor cells (4T1 mouse mammary; B16 mouse melanoma; mouse Lewis Lung Carcinoma; CT26 mouse colorectal; BT549 human TNBC) were treated with vehicle control, neratinib (0.5 μM), sodium valproate (250 μM) or the drugs in combination. Twenty-four h after drug exposure, cells were treated with live/dead reagent and cell viability determined as described in the Methods. (n = 3 +/-SEM) ^#^ p < 0.05 greater than corresponding value in neratinib single agent cells.

As presented in Figure 1A, the expression of the non-receptor tyrosine kinase c-SRC was modestly reduced in the afatinib resistant clones. Previously, we have demonstrated that afatinib resistant clones exhibit higher c-SRC Y416 and lower c-SRC Y527 phosphorylation than the parental clones, collectively indicative of c-SRC activation [1]. Treatment of the afatinib resistant clones with sodium valproate or AR42 further activated c-SRC as judged by the phosphorylation of c-SRC Y527 decreasing and the phosphorylation of c-SRC Y416 increasing (Figure 2A). The receptors ERBB1 and ERBB3 can be phosphorylated by c-SRC, which subsequently leads to full receptor activation [22]. Treatment of afatinib resistant clones with sodium valproate reduced total ERBB1 expression in 4/5 clones but increased ERBB1 Y1068/Y1173 phosphorylation in all clones by approximately 2-3-fold (Figure 2B). Similar findings were also made for ERBB3 expression and phosphorylation. These observations prompted us to test the hypothesis that HDAC inhibitors will interact with neratinib to enhance tumor cell killing.

Neratinib is a third-generation orally available ERBB1/2/4 inhibitor that irreversibly binds to ERBB1, ERBB2 and ERBB4. Neratinib has been shown to have clinical activity in breast cancers with ERBB2 amplifications or mutations. Neratinib significantly enhanced valproate lethality in multiple tumor cell types, including PDX models of mutant B-RAF melanoma, glioblastoma and ovarian cancer, in colon and pancreatic cancer, and in the afatinib resistant H1975 clones (Figure 2C; Table 1 ; Supplementary Figure 2). Of note, whilst both neratinib and afatinib were competent as single agents to kill 5/5 parental wild type H1975 clones, only neratinib could kill 5/5 afatinib resistant H1975 clones, both as a single agent and when combined with valproate (Table 1). The second generation ERBB1/2/4 inhibitor lapatinib did not impact on tumor cell viability in the afatinib resistant clones. The E3 ligase NEDD4 regulates the expression of the lipid phosphatase PTEN [23]. Knock down of NEDD4 enhanced PTEN expression and enhanced the ability of neratinib to kill afatinib resistant H1975 clones (Supplementary Figure 3). Collectively, our findings argue that neratinib can negate the HDAC inhibitor -induced activation of c-SRC / ERBB1 / ERBB3 / AKT survival signaling, thus enhancing cell killing by HDAC inhibitors, and as a single agent that neratinib can overcome afatinib resistance in 5/5 afatinib resistant H1975 clones.

**Table 1:** Neratinib kills afatinib resistant H1975 clones and interacts with valproate to enhance tumor cell killing

	VEH	NER	LAP	AFA	VEH	NER	LAP	AFA	VEH	NER	LAP	AFA	VEH	NER	LAP	AFA
% death	1	12	2	14	3	17	6	15	1	23^##^	2	5¶	10	39^###^	8	9
% death	1	14	2	19	2	26^#^	8	16	1	35^##^	3	6¶	10	50^###^	14	13
% death	1	17	2	12	2	18	7	29^#^	1	38^##^	6	3¶	13	61^###^	19	14
% death	1	24	8	13	2	28	10	11	1	30^##^	8	4¶	9	75^###^	18	7
% death	1	18	2	11	3	39^#^	14^#^	21^#^	1	25^##^	10	4¶	11	47^###^	16	15
VEH						VAL			VEH				VAL			
Parental H1975 clones									Afatinib-R H1975 clones							

Parental and afatinib resistant H1975 clones were treated with vehicle control, sodium valproate (250 μM), neratinib (0.5 μM), afatinib (0.5 μM), lapatinib (2.0 μM) or the drugs in combination as indicated in the Table. Twenty-four h after drug exposure, cells were treated with live/dead reagent and cell viability determined as described in the Methods. (n = 3 +/-SEM) ^#^ p < 0.05 greater than corresponding value in vehicle control cells; ^##^ p < 0.05 greater than corresponding value in parental H1975 clones; ^###^ p < 0.05 greater value than corresponding value in afatinib resistant clones; ¶ p < 0.05 less than corresponding value in parental H1975 clones

ERBB family receptors can both homo- and hetero-dimerize. In the case of our afatinib resistant clones, ERBB1 can homo-dimerize with itself and hetero-dimerize with ERBB2, ERBB3 and ERBB4. ERBB4 can homo-dimerize with itself and hetero-dimerize with ERBB1, ERBB2 and ERBB3. ERBB3 cannot homo-dimerize. In parental wild type H1975 clones, ERBB1 and ERBB3 did not appear to strongly co-localize as judged by the separate red and green staining profiles (Supplementary Figure 4). In the afatinib resistant clones, in addition to separate red and green staining were also areas of yellow, indicating a co-localization of ERBB1 and ERBB3. In contrast to data with ERBB1 and ERBB3, ERBB1 and ERBB4 exhibited co-localization in both parental and afatinib resistant clones (Supplementary Figure 5). The co-localization of c-SRC and ERBB1 was very similar in parental and afatinib resistant clones (Supplementary Figure 6). In afatinib resistant clones we demonstrated that sodium valproate activated c-SRC, that correlated with increase co-localization of PI3K p110α/β with ERBB3 (Supplementary Figure 7). Valproate increased the co-localization of ERBB1 and ERBB3, but not of ERBB1 and ERBB4 where the co-localization of the receptors in some clones declined (Supplementary Figures 8 and 9).

**Figure 3:**
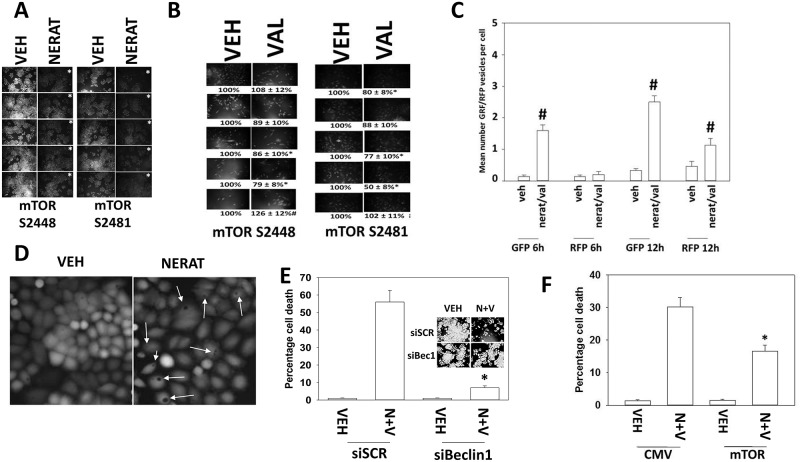
Neratinib lethality requires autophagosome formation **(A)** Afatinib resistant clones were treated with vehicle control or with neratinib (0.5 μM). After 6h cells were fixed in place and immunofluorescent staining performed to detect the phosphorylation of mTOR S2448 (mTORC1) and mTOR S2481 (mTORC2). (n = 3 +/-SEM) ^*^ p < 0.05 lower staining intensity than in vehicle control treated clones. **(B)** Afatinib resistant clones were treated with vehicle control or with sodium valproate (250 μM). After 6h cells were fixed in place and immunofluorescent staining performed to detect the phosphorylation of mTOR S2448 (mTORC1) and mTOR S2481 (mTORC2). (n = 3 +/-SEM) ^*^ p < 0.05 lower staining intensity than in vehicle control treated clones; ^#^ p < 0.05 higher staining intensity than in vehicle control treated clones. **(C)** Afatinib resistant clones were transfected with a plasmid to express LC3-GFP-RFP. Twenty-four h after transfection cells were treated with vehicle control or with [neratinib (0.5 μM) + valproate (250 μM)] for 6h and for 12h. The number of intense staining GFP+ and RFP+ foci was determined from 40 cells per condition. (n = 3 +/-SEM) ^#^ p < 0.05 greater than vehicle control. **(D)** An afatinib resistant clone was treated with vehicle control or with neratinib for 24h. Cells were stained with live/dead reagent and images at 10X magnification obtained. Arrows indicate the presence of large vesicle structures in the cells. **(E)** Afatinib resistant clones were transfected with a scrambled control siRNA or with an siRNA to knock down Beclin1. Twenty-four h later, cells were treated with vehicle control or with [neratinib (0.5 μM) + valproate (250 μM)]. After an additional 24h cells were treated with live/dead reagent and the percentage cell death under each condition determined. Data are the mean death from the 5 clones. (n = 3 +/-SEM) ^*^ p < 0.05 less killing compared to siSCR cells. The representative inset panel shows total cell numbers. **(F)** Afatinib resistant clones were transfected with an empty vector plasmid or a plasmid to express an activated form of mTOR. Twenty-four h later, cells were treated with vehicle control or with [neratinib (0.5 μM) + valproate (250 μM)]. After an additional 24h cells were treated with live/dead reagent and the percentage cell death under each condition determined. (n = 3 +/-SEM) ^*^ p < 0.05 less killing compared to siSCR cells.

We next determined the mechanisms by which neratinib kills afatinib resistant H1975 cells. Neratinib inactivated mTORC1 (S2448) and mTORC2 (S2481) in 5/5 clones, each by ~50% (Figure 3A). The trend for sodium valproate was to modestly reduce mTORC1 and mTORC2 activity (Figure 3B). Treatment of cells with [neratinib + valproate] increased the numbers of autophagosomes, and subsequently the number of autolysosomes in drug-treated cells (Figure 3C). Knock down of Beclin1, ATG5 or ULK1 did not prevent neratinib from inactivating mTORC1 and mTORC2 (data not shown). In cells treated with neratinib, the rapid appearance of large intracellular vesicles, more reminiscent of plant vacuoles than autophagosomes, was observed (Figure 3D). Knock down of Beclin1 suppressed killing by [neratinib + valproate] and prevented the profound reduction in total cell numbers (Figure 3E). Expression of an activated form of mTOR prevented autophagosome formation and reduced [neratinib + valproate] lethality (Figure 3F, not shown). Collectively our data argue that inactivation of mTOR and the induction of autophagic flux play a major role in the anti-tumor activity of neratinib and the [neratinib + valproate] drug combination.

**Figure 4:**
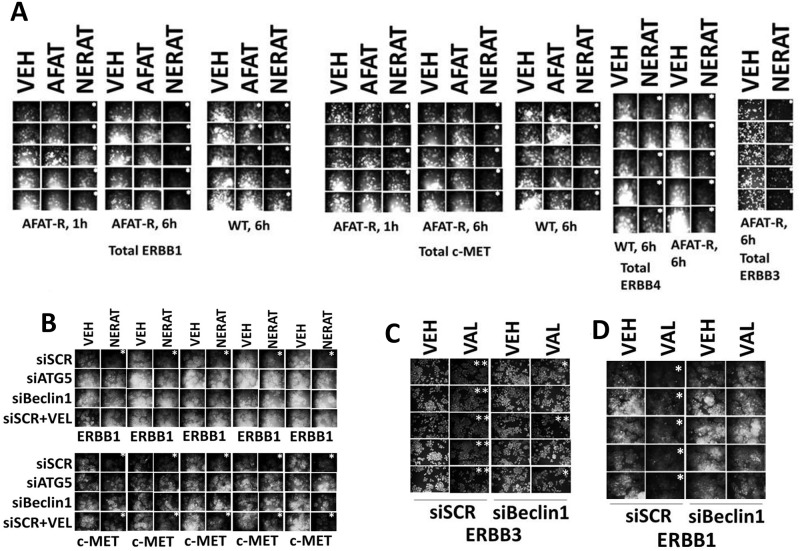
Neratinib reduces the expression of ERBB receptors and c-MET via autophagic degradation **(A)** Wild type parental and afatinib resistant H1975 clones were treated with vehicle control, afatinib (0.5 μM) or neratinib (0.5 μM). After 6h cells were fixed in place and immunostaining performed to detect the total expression of ERBB1, ERBB3, ERBB4 and c-MET. (n = 3 +/-SEM) ^*^ p < 0.05 less intensity of staining compared to vehicle control cells. **(B)** Afatinib resistant H1975 clones were transfected with a scrambled control siRNA or with siRNA molecules to knock down ATG5 or Beclin1. Twenty-four h after transfection cells were pre-treated for 1h with vehicle control or Velcade (10 nM) and then treated with vehicle control or with neratinib (0.5 μM). After 6h cells were fixed in place and immunostaining performed to detect the total expression of ERBB1 and c-MET. (n = 3 +/-SEM) ^*^ p < 0.05 less intensity of staining compared to vehicle control cells. **(C)** and **(D)** Afatinib resistant H1975 clones were transfected with a scrambled control siRNA or with an siRNA molecule to knock down Beclin1. Twenty-four h after transfection cells were treated with vehicle control or with sodium valproate (250 μM). After 6h cells were fixed in place and immunostaining performed to detect the total expression of ERBB1 and ERBB3. (n = 3 +/-SEM) ^*^ p < 0.05 less intensity of staining compared to corresponding vehicle control cells; ^**^ p < 0.01 less intensity of staining compared to corresponding vehicle control cells.

Unlike neratinib, neither afatinib nor lapatinib as single agents caused the appearance of the large intracellular “vacuoles” in tumor cells (Figure 3D, data not shown). Yet, all three drugs act upon cells by inhibiting ERBB1/2/4. We reasoned that other than the efficacy / concentrations at which they inhibit ERBB1/2/4, the only major difference in molecular action between neratinib, afatinib and lapatinib is that neratinib chemically modifies ERBB1/2/4 as part of its inhibitory effect, i.e. neratinib is an irreversible inhibitor. As such, we compared the impact of neratinib and of afatinib on the total protein expression of ERBB1, ERBB3, ERBB4 and as a control c-MET in the afatinib resistant clones. In a time-dependent fashion, neratinib, but not afatinib, reduced the total expression of ERBB1, ERBB3 and ERBB4 (Figure 4A). In cells not expressing a mutated active ERBB family receptor neratinib, but not afatinib, rapidly reduced expression of ERBB1 and ERBB2 (Supplementary Figure 10).

Studies with the receptor c-MET were originally performed to act as a negative control, i.e. neratinib does not inhibit or chemically modify c-MET. However, to our surprise, we discovered that neratinib, but not afatinib, down-regulated c-MET expression. The reasons why neratinib could so rapidly reduce c-MET expression, e.g. internalization of quaternary receptor tyrosine kinase signalosomes, will require studies beyond the scope of the present manuscript. Knock down of the autophagy regulatory proteins Beclin1 or ATG5, or treatment with the E3 ligase inhibitor bortezomib (Velcade) prevented the down-regulation of ERBB1. In contrast, knock down of Beclin1 or ATG5 prevented c-MET down regulation, but treatment of the cells with the proteasome inhibitor did not (Figure 4B). Sodium valproate was also capable of reducing the total expression of ERBB1 and ERBB3, and effect that was prevented by knock down of Beclin1 (Figure 4C and 4D). Thus, neratinib both inhibits and down-regulates the expression ERBB family receptor tyrosine kinases as well as other RTKs.

**Figure 5:**
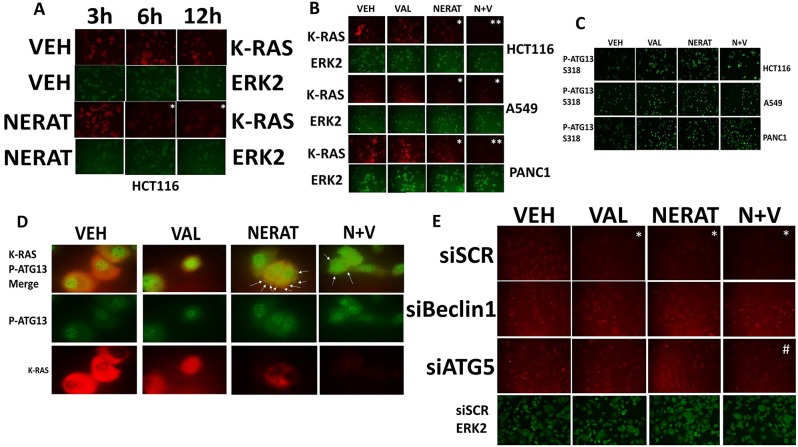
Neratinib down-regulates the expression of K-RAS in tumor cells expressing mutated active K-RAS **(A)** HCT116 colon cancer cells that express a mutant active K-RAS were treated with vehicle control or with neratinib (0.5 μM) for up to 12h. Cells were fixed in place at each time point and immunostaining performed to determine the expression of K-RAS. (n = 3 +/-SEM) ^*^ p < 0.05 less staining intensity than corresponding vehicle control value. **(B)** HCT116 (colon); A549 (NSCLC); PANC-1 (pancreatic) tumor cells were treated with vehicle control, sodium valproate (250 μM), neratinib (0.5 μM) or the drugs combined for 6h. Cells were fixed in place and immunostaining performed to determine the expression of K-RAS. (n = 3 +/-SEM) ^*^ p < 0.05 less than corresponding vehicle control value; ^**^p < 0.05 less than intensity in neratinib treated cells. **(C)** HCT116 (colon); A549 (NSCLC); PANC-1 (pancreatic) tumor cells were treated with vehicle control, sodium valproate (250 μM), neratinib (0.5 μM) or the drugs combined for 6h. Cells were fixed in place and immunostaining performed to determine the phosphorylation of ATG13 S318. **(D)** PANC-1 cells were treated with vehicle control, sodium valproate (250 μM), neratinib (0.5 μM) or the drugs combined for 6h. Cells were fixed in place and immunostaining performed to determine the co-localization of K-RAS with P-ATG13 S318 at 60X magnification. **(E)** PANC-1 cells were transfected with a scrambled siRNA control, or with siRNA molecules to knock down the expression of Beclin1 or ATG5. Twenty-four h after transfection cells were treated with vehicle control, sodium valproate (250 μM), neratinib (0.5 μM) or the drugs combined for 6h. Cells were fixed in place and immunostaining performed to determine the expression of K-RAS. (n = 3 +/-SEM) ^*^ p < 0.05 less than corresponding vehicle control value; ^#^p < 0.05 greater than intensity of corresponding treatment in siSCR cells.

As we were observing reductions in ERBB family receptors and c-MET, we reasoned that other membrane-associated signaling proteins may also have their expression levels reduced by neratinib. The proto-oncogene K-RAS is often mutated in lung, pancreatic and colon cancer. Signaling by mutant K-RAS into the ERK1/2, ERK5, JNK and PI3K pathways plays key roles in the transforming, growth promoting and apoptosis-resistant phenotype of mutant K-RAS expressing tumor cells. Neratinib treatment, in addition to reducing the expression of receptor tyrosine kinases also reduced the expression of mutated active K-RAS in multiple tumor cell types (Figure 5A and 5B). Neratinib and sodium valproate interacted to further reduce K-RAS expression that was associated with increased ATG13 S318 phosphorylation (Figure 5B and 5C). At 60X magnification, phosphorylated ATG13 S318 co-localized with K-RAS in large vesicular structures (Figure 5D, white arrows). Knock down of the autophagy regulatory proteins ATG5 or Beclin1 prevented neratinib from reducing K-RAS expression (Figure 5E). Of note was that knock down of ATG5 was not as effective as knock down of Beclin1 at preventing the [neratinib + valproate] -induced down-regulation of K-RAS. In an ovarian cancer cell line expressing a mutant N-RAS, neratinib, and to a greater extent [neratinib + valproate] reduced N-RAS expression in an autophagy-dependent fashion (Supplementary Figure 11). Collectively our data argue that neratinib may have efficacy in treating tumors expressing mutant K-RAS or mutant N-RAS.

**Figure 6:**
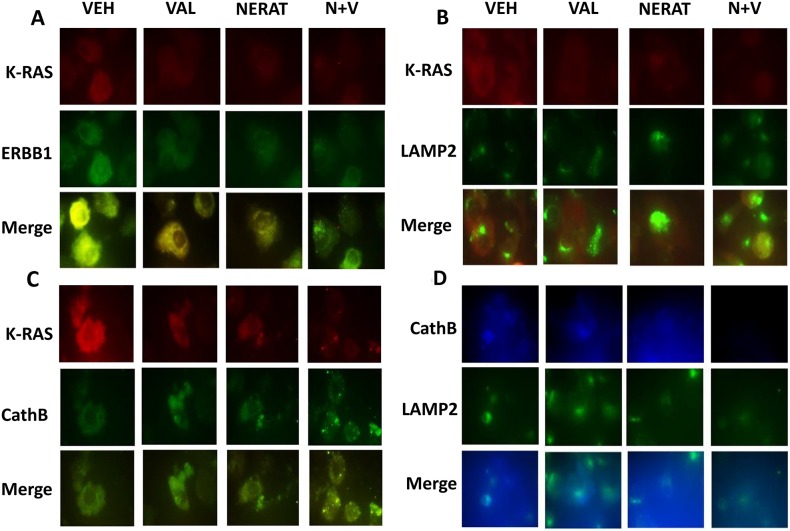
Neratinib promotes the co-localization of K-RAS with LAMP2 and cathepsin B, and the disassociation of K-RAS and ERBB1 **(A)** PANC-1 cells were treated with vehicle control, sodium valproate (250 μM), neratinib (0.5 μM) or the drugs combined for 6h. Cells were fixed in place and immunostaining performed to determine the co-localization of K-RAS with ERBB1 at 60X magnification. **(B)** PANC-1 cells were treated with vehicle control, sodium valproate (250 μM), neratinib (0.5 μM) or the drugs combined for 6h. Cells were fixed in place and immunostaining performed to determine the co-localization of K-RAS with LAMP2 at 60X magnification. **(C)** PANC-1 cells were treated with vehicle control, sodium valproate (250 μM), neratinib (0.5 μM) or the drugs combined for 6h. Cells were fixed in place and immunostaining performed to determine the co-localization of K-RAS with cathepsin B at 60X magnification. **(D)** PANC-1 cells were treated with vehicle control, sodium valproate (250 μM), neratinib (0.5 μM) or the drugs combined for 6h. Cells were fixed in place and immunostaining performed to determine the co-localization of LAMP2 and cathepsin B at 60X magnification.

Based on our co-localization studies in Figure 5, using the PANC-1 pancreatic carcinoma cell line which expresses higher levels of K-RAS than the A549 NSCLC line, we determined whether our drug treatments altered the co-localization of K-RAS with ERBB1, and with the lysosomal associated proteins LAMP2 and cathepsin B. ERBB1 and K-RAS co-localized in PANC-1 cells, which was disrupted by exposure to neratinib, valproate and the drug combination (Figure 6A). Neratinib, and to a greater extent [neratinib + valproate], promoted co-localization of K-RAS with LAMP2 or with cathepsin B (Figure 6B and 6C). Based on the co-localization images in Figure 6B and 6C being dissimilar, we also examined whether LAMP2 and cathepsin B co-localized. Although co-localization of LAMP2 and cathepsin B was observed, it was evident that there are intracellular pools of LAMP2 and cathepsin B that do not co-localize. The origin and processing of K-RAS following neratinib exposure will require studies beyond the scope of the present manuscript.

**Figure 7:**
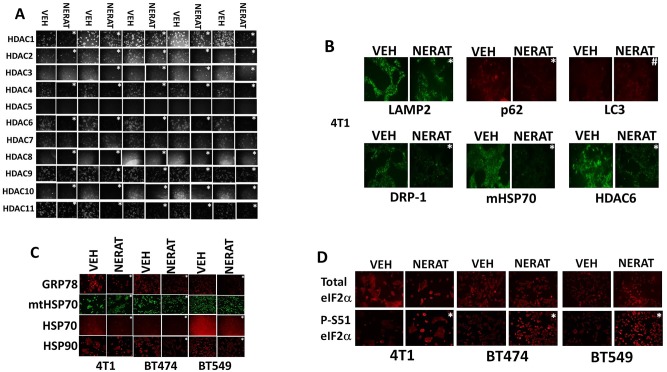
Neratinib regulates the expression of HDAC proteins and generates an endoplasmic reticulum stress response **(A)** Afatinib resistant H1975 clones were treated with vehicle control or with neratinib (0.5 μM) for 6h. Cells were fixed in place and immunostaining performed to determine the expression of HDACs1-11. (n = 3 +/-SEM) ^*^ p < 0.05 less staining intensity than corresponding vehicle control value. **(B) (C and D)** Mouse TNBC cells (4T1), human BT474 HER2+ and BT549 TNBC cells, were treated with vehicle control or with neratinib (0.5 μM) for 6h. Cells were fixed in place and immunostaining performed to determine the expression of eIF2α, ERK1/2, LAMP2, p62 SQMT1, LC3, DRP-1, HDAC6, mitochondrial HSP70, HSP70, HSP90 and GRP78, and the phosphorylation of eIF2α S51. (n = 3 +/-SEM) ^*^ p < 0.05 less staining intensity than corresponding vehicle control value.

We have recently published that histone deacetylase proteins can be down-regulated via autophagic digestion. Hence, we hypothesized that in addition to reducing the expression of receptors and K-RAS, treatment of the afatinib resistant H1975 clones with neratinib would reduce the expression of multiple HDAC proteins; the hypothesis was correct and we observed neratinib reducing the levels of HDAC1, HDAC2, HDAC3, HDAC6 and HDAC10 (Figure 7A). Treatment TNBC and ERBB2+ breast cancer cells with neratinib also reduced the expression of p62 SQMT1, LAMP2, HDAC6, DRP-1, mitochondrial HSP70, HSP70, HSP90 and GRP78 (Figure 7B and 7C). The expression of LC3 was also modestly enhanced. In agreement with reduced GRP78 expression, neratinib also enhanced eIF2α S51 phosphorylation, i.e. neratinib is enhancing autophagic flux and generating an endoplasmic reticulum stress response (Figure 7D).

Over the past five years, multiple studies from this laboratory have demonstrated that inhibitors of ERBB1/2/4 (lapatinib, afatinib) can enhance the lethality of cytotoxic drugs and other kinase inhibitors. Neratinib enhanced the lethality of [pemetrexed + sorafenib] *in vitro* and *in vivo* (Supplementary Figures 12 and 13); of [regorafenib + sildenafil] (Supplementary Figure 14); of dasatinib (Supplementary Figure 15); and of ruxolitinib (Supplementary Figure 16A) [1, 24–27]. We have recently published studies demonstrating that HDAC inhibitors can enhance the lethality of dabrafenib / trametinib in PDX B-RAF mutant melanoma isolates [28]. In all mutant B-RAF isolates tested, neratinib profoundly enhanced the lethality of dabrafenib / trametinib (Supplementary Figure 16B). The *in vivo* data in Supplementary Figure 13 confirms prior *in vivo* studies using lapatinib and afatinib in combination with [pemetrexed + sorafenib], demonstrating that transient inhibition of ERBB1/2/4 significantly reduced tumor growth in the presence of [pemetrexed + sorafenib]. As the open phase II trial of [pemetrexed + sorafenib] already has several TNBC patients with a confirmed PR or prolonged SD response, these findings further validate initiating a new phase I trial combining [pemetrexed + sorafenib + neratinib] [29].

The treatment of NSCLC has been revolutionized using checkpoint inhibitory antibodies [30]. It is known that patients whose mutant ERBB1 expressing tumors become resistant to ERBB inhibitors have a poorer response to checkpoint inhibitory antibodies than patients with other genetic NSCLC variants [31]. In general agreement with those findings, afatinib-resistant H1975 clones expressed lower levels of PD-L1, PD-L2, MHCA and HMGB1, and enhanced levels of ornithine decarboxylase (ODC) compared to the parental clones (Supplementary Figure 17A). Treatment of a genetically diverse set of NSCLC lines with valproate reduced the expression of PD-L1, PD-L2 and ODC, and increased the expression of MHCA and HMGB1 (Supplementary Figure 17B). In the afatinib resistant H1975 clones, valproate also reduced PD-L1, PD-L2 and ODC levels and increased MHCA expression (Supplementary Figure 17C). Based on this data, and the fact that afatinib resistant clones over-expressed HDAC3 and HDAC10, we determined whether either or both HDACs regulated the expression of the immunogenic biomarkers. Knock down of HDAC3 in a clonal dependent fashion reduced the expression of PD-L1 and PD-L2 and enhanced MHCA levels (Supplementary Figure 17D). HDAC10 knock down reduced PD-L1 and ODC expression, and enhanced MHCA levels. Combined knock down of HDAC3 and HDAC10 facilitated a further decline in ODC expression.

**Figure 8:**
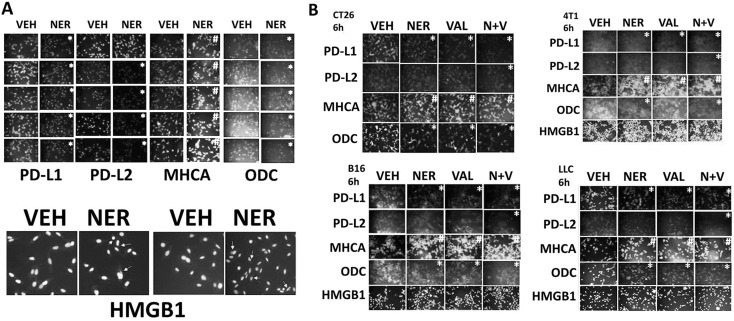
Neratinib regulates the expression of immunotherapy biomarkers **(A)** Afatinib resistant clones were treated for 6h with vehicle control or with neratinib (0.5 μM). Cells were then fixed in place and immunostaining performed to determine the expression levels of PD-L1, PD-L2, MHCA, ODC, HMGB1. (n = 3 +/-SEM) ^*^ p < 0.05 less intensity of staining compared to vehicle control cells; ^#^ p < 0.05 greater intensity of staining compared to vehicle control cells. **(B)** Tumor cells (CT26 mouse colorectal; 4T1 mouse mammary; B16 mouse melanoma; mouse Lewis Lung Carcinoma) were treated for 6h with vehicle control, neratinib (0.5 μM), sodium valproate (250 μM) or the drugs in combination. Cells were then fixed in place and immunostaining performed to determine the expression levels of PD-L1, PD-L2, MHCA, ODC, HMGB1. (n = 3 +/-SEM) ^*^ p < 0.05 less intensity of staining compared to vehicle control cells; ^#^ p < 0.05 greater intensity of staining compared to vehicle control cells.

We then investigated whether the drug combination of [neratinib + valproate] could further affect the immunogenicity profile of afatinib-resistant H1975 clones. To this end, we measured the impact of neratinib on the expression of PD-L1, PD-L2, MHCA, ODC and HMGB1. In afatinib resistant H1975 clones, neratinib, as a single agent, reduced the expression of PD-L1, PD-L2 and ODC, and increased the levels of MHCA (Figure 8A). Neratinib also caused the extracellular release of HMGB1. In spontaneous mouse colorectal, mammary, lung and breast tumor isolates, both neratinib and valproate, alone or in combination, reduced the expression of PD-L1, PD-L2 and ODC and enhanced the expression of MHCA (Figure 8B). Similar findings were made in human mammary BT549 cells (Supplementary Figure 18). The expression of PD-L1, PD-L2 and ODC was reduced and the levels of MHCA enhanced after exposure of tumor cells to [pemetrexed + sorafenib], [regorafenib + sildenafil], [neratinib + dasatinib] and [ruxolitinib + neratinib] (Supplementary Figures 19-23). Collectively, the data in Figures 6-8 and in the supplemental data argues that [neratinib + valproate] treatment has the potential to sensitize tumor cells to T cell mediated killing by increasing the levels of MHC class I on the tumor surface and by reducing the expression of inhibitory ligands such as PD-L1.

**Figure 9:**
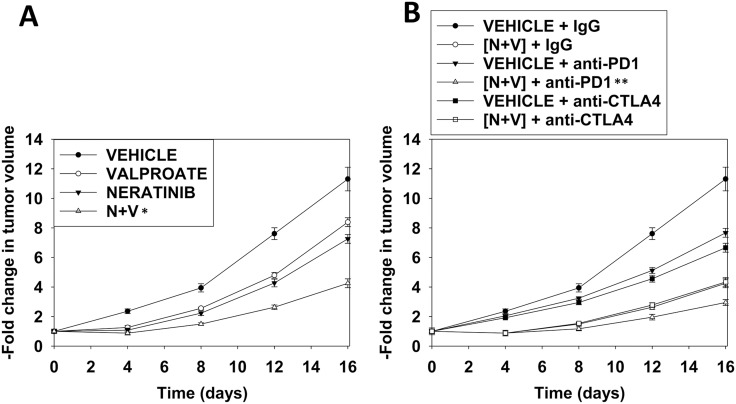
Neratinib and valproate interact to suppress tumor growth and to opsonize the surviving tumor cells to checkpoint immunotherapies **(A)** and **(B)** BALB/c mice were implanted with 4T1 cells in the 4^th^ mammary fat pad and ~30 mm^3^ tumors permitted to form. Animals were then treated with vehicle control, neratinib (15 mg/kg QD), valproate (50 mg/kg BID) or the drugs in combination for 3 days. Two days after the cessation of drug exposure mice were injected IP with a control IgG (100 μg / mouse); an anti-PD-1 antibody (100 μg / mouse); or an anti-CTLA4 antibody (100 μg / mouse). Tumor volumes were measured prior to drug administration and every three days after the initiation of therapeutic interventions. (n = 10 mice per group +/-SEM). ^*^ p < 0.05 less than neratinib alone or valproate alone; ^**^ p < 0.05 less than IgG + [neratinib + valproate].

As neratinib is approved for the treatment of breast cancer, we chose to perform definitive animal studies in the highly aggressive 4T1 TNBC mammary carcinoma isolate [25]. Small 4T1 tumors were formed in the 4^th^ mammary fat pads of syngeneic BALB/c mice and the animals treated with neratinib and valproate followed by treatment with anti-PD-1 or anti-CTLA4 antibodies. Neratinib and sodium valproate combined in an at least additive fashion to suppress mammary tumor growth (Figure 9A). The anti-tumor effects of [neratinib + valproate] exposure was amplified by a subsequent administration of an anti-PD-1 antibody but not by an anti-CTLA4 antibody (Figure 9B). Collectively our findings validate the concept that neratinib and HDAC inhibitors combine to both kill mammary tumor cells *in vivo* and to sensitize the remaining cells to checkpoint inhibitory immunotherapy.

## DISCUSSION

The present studies were designed to further investigate the activity of neratinib either alone or in combination with other drugs. The key discoveries made in this manuscript are that neratinib at clinically relevant concentrations kills afatinib resistant NSCLC cells and such lethality can be enhanced by HDAC inhibitors. In addition, neratinib / HDAC inhibitor combination therapy enhances the immunogenicity profile of tumor cells possibly unveiling as sensitivity of the remaining tumor cells to immunotherapy agents, such as immune-checkpoint inhibition.

In addition to the primary goal of these studies, we also confirmed that neratinib was as effective as afatinib or lapatinib at enhancing the lethality of the previously established drug combinations of [pemetrexed + sorafenib], [regorafenib + sildenafil], [neratinib + dasatinib] and [ruxolitinib + neratinib]. These combinations also altered the expression of immunoregulatory proteins such that it would be predicted that these drug combinations could synergize with checkpoint immunotherapy antibodies. Based on prior studies, the ability of neratinib to enhance the anti-tumor efficacy of [pemetrexed + sorafenib] *in vivo* trended to be greater than the ability of afatinib. We are presently developing a protocol to perform a three drug all solid tumor phase I trial in 2018 combining [pemetrexed + sorafenib + neratinib].

One unexpected observation from our studies was that neratinib caused the breakdown of ERBB1, ERBB3, ERBB4 and c-MET through an autophagy-dependent process. It is known that the ERBB1 ligands EGF and TGFα differentially regulate the signaling, internalization and recycling of the receptor [32–34]. EGF remains attached to ERBB1 in the acidic endosome environment which leads to an initial prolonged signaling response but that subsequently results in receptor degradation. On the other hand, TGFα dissociates from the internalized ERBB1 resulting in receptor inactivation and receptor recycling to the cell surface. These processes can also result in a differential biologic response of cells to EGF exposure [35]. Squamous A431 carcinoma cells treated with 0.1-0.5 ng/ml of EGF proliferate; cells treated with 1-2 ng/ml EGF growth arrest; and cells treated with 5-10 ng/ml EGF undergo apoptosis within 24h. The precise mechanisms by which neratinib induces receptor tyrosine kinase internalization and degradation are presently unknown. That an ERBB1/2/4 specific inhibitor reduced the expression of a receptor, c-MET, that is not catalytically inhibited by neratinib, and the kinase dead receptor ERBB3 that does not bind ATP, argues that the chemical modification of ERBB1/2/4 must trigger a seismic event in the plasma membrane where not only are ERBB family receptors internalized but “fellow-traveler” receptor tyrosine kinases are also routed to lysosomal degradation. Studies beyond the scope of the present manuscript will be required to fully understand this novel component of neratinib biology.

One reason why ERBB1 specific inhibitors have proved less effective in the clinic compared to inhibitors which block ERBB1/2/4 is that the ERBB family of receptors can heterodimerize with each other [33]. Neither gefitinib nor erlotinib can prevent ERBB2 from trans-phosphorylating ERBB3, thereby potentially facilitating an evolutionary survival pathway. Parental and afatinib resistant H1975 cells express low levels of ERBB2 that decline in the afatinib resistant clones [1]. In our afatinib resistant clones we were already cognascent that c-SRC was activated, and in these clones whilst we found that although the localization of c-SRC with ERBB1 was not altered by afatinib resistance, the c-SRC targets ERBB1 and ERBB3 became co-localized [1]. The survival role for signaling through ERBB3 had already been established for the afatinib resistant clones and in the afatinib resistant clones we found that the further activation of c-SRC by valproate increased the co-localization of PI3K p110α/β with ERBB3. It has been shown by others that c-SRC can also facilitate the activation of c-MET, collectively arguing for our system that the evolution of c-SRC activation in the afatinib resistant H1975 clones is a primary evolutionary survival event [36]. The molecular mechanisms by which c-SRC becomes activated in the afatinib resistant clones is at present unknown.

Recent prior studies from our laboratory have demonstrated that drug combinations which induce autophagosome and autolysosome formation, for example [pemetrexed + sildenafil] or [pazopanib + HDAC inhibitors], can reduce the expression of HDACs, particularly HDAC6 [28, 37–40]. HDAC6 is a cytosolic HDAC that regulates the activities of HSP90 and HSP70, and inhibition of HDAC6 function reduces the chaperoning functions of these proteins promoting endoplasmic reticulum stress and autophagy [41, 42]. Neratinib as a single agent reduced the expression of HDAC6 and enhanced the expression of Beclin1, effects that were magnified by valproate. Neratinib also reduced the expression of HDACs2/4/10 which argues that neratinib-dependent reductions in HDAC10 levels will directly impact on the expression of immunotherapeutic biomarkers. The HDAC-dependent changes in tumor cell biology caused by drugs that are themselves not HDAC inhibitors will require studies beyond the scope of the present paper.

Immunotherapy is a standard of care modality in NSCLC and is now approved to be combined with pemetrexed and carboplatin as a 1^st^ line therapy. The 1^st^ line therapy for NSCLC patients who express a mutated active form of ERBB1 is an ERBB1 inhibitor (erlotinib, gefitinib, afatinib). Over the next 6-18 months the NSCLC tumors evolve so that they become resistant to the kinase inhibitory drugs. It is known from *in vitro* studies that ERBB1 inhibitors reduce the expression of immunotherapy biomarkers such as PD-L1 in NSCLC cells and tumors from kinase inhibitor resistant patients express low levels of PD-L1 and PD-L2 [43, 44, 45–46]. In NSCLC, low levels of immunotherapy biomarkers correlate with a poor anti-tumor response to anti-PD-1 and anti-CTLA4 inhibitory antibodies. Our findings argue that not only can [neratinib + valproate] kill afatinib resistant tumor cells, but it can also sensitize them to checkpoint inhibitory antibodies which facilitate immunological tumor cell destruction. Only clinical studies in afatinib-resistant patients will prove or refute whether the present findings will translate into better outcomes and survival.

## MATERIALS AND METHODS

### Materials

Sodium valproate was from Sigma (St. Louis, MO). Neratinib was supplied by Puma Biotechnology Inc. (Los Angeles, CA). Sorafenib tosylate, dasatinib, ruxolitinib, dabrafenib, trametinib and sildenafil were from Selleckchem (Houston TX). Trypsin-EDTA, DMEM, RPMI, penicillin-streptomycin were purchased from GIBCOBRL (GIBCOBRL Life Technologies, Grand Island, NY). All “H” series NSCLC lines were purchased from the ATCC and were not further validated beyond that claimed by ATCC. Cells were re-purchased every ~6 months. ADOR cells were a gift to the Dent lab from a female NSCLC patient. Spiky ovarian cancer cells were kindly provided by Dr. Karen Paz (Champions Oncology, NJ). Commercially available validated short hairpin RNA molecules to knock down RNA / protein levels were from Qiagen (Valencia, CA) (Supplementary Figure 24). Control IgG, anti-PD-1 and anti-CTLA4 endotoxin-free antibodies were purchased from Bio-X cell (West Lebanon, NH). Reagents and performance of experimental procedures were described in refs: 1, 24-28, 45, 46.

### Methods

#### Culture and *in vitro* exposure of cells to drugs

All cell lines were cultured at 37 °C (5% (v/v CO_<sub>2</sub>_) *in vitro* using RPMI supplemented with dialyzed 5% (v/v) fetal calf serum and 10% (v/v) Non-essential amino acids. For short term cell killing assays, immune-staining studies, cells were plated at a density of 3 × 10^3^ per cm^2^ and 24h after plating treated with various drugs, as indicated. *In vitro* drug treatments were generally from a 100 mM stock solution of each drug and the maximal concentration of Vehicle carrier (VEH; DMSO) in media was 0.02% (v/v). Cells were not cultured in reduced serum media during any study in this manuscript.

### Transfection of cells with siRNA or with plasmids

#### For Plasmids

Cells were plated and 24h after plating, transfected. Plasmids expressing a specific mRNA (or siRNA) or appropriate vector control plasmid DNA was diluted in 50μl serum-free and antibiotic-free medium (1 portion for each sample). Concurrently, 2μl Lipofectamine 2000 (Invitrogen), was diluted into 50μl of serum-free and antibiotic-free medium (1 portion for each sample). Diluted DNA was added to the diluted Lipofectamine 2000 for each sample and incubated at room temperature for 30 min. This mixture was added to each well / dish of cells containing 200μl serum-free and antibiotic-free medium for a total volume of 300 μl, and the cells were incubated for 4 h at 37 °C. An equal volume of 2x medium was then added to each well. Cells were incubated for 24h, then treated with drugs.

#### Transfection for siRNA

Cells from a fresh culture growing in log phase as described above, and 24h after plating transfected. Prior to transfection, the medium was aspirated and serum-free medium was added to each plate. For transfection, 10 nM of the annealed siRNA, the positive sense control doubled stranded siRNA targeting GAPDH or the negative control (a “scrambled” sequence with no significant homology to any known gene sequences from mouse, rat or human cell lines) were used. Ten nM siRNA (scrambled or experimental) was diluted in serum-free media. Four μl Hiperfect (Qiagen) was added to this mixture and the solution was mixed by pipetting up and down several times. This solution was incubated at room temp for 10 min, then added drop-wise to each dish. The medium in each dish was swirled gently to mix, then incubated at 37 °C for 2h. Serum-containing medium was added to each plate, and cells were incubated at 37 °C for 24h before then treated with drugs (0-24h). Additional immuno-fluorescence / live-dead analyses were performed at the indicated time points.

#### Detection of cell viability, protein expression and protein phosphorylation by immuno-fluorescence using a Hermes WiScan machine

http://www.idea-bio.com/, Cells (4 × 10^3^) are plated into each well of a 96 well plate, and cells permitted to attach and grow for the next 18h. Based on the experiment, after 18h, cells are then either genetically manipulated, or are treated with drugs. For genetic manipulation, cells are transfected with plasmids or siRNA molecules and incubated for an additional 24h. Cells are treated with vehicle control or with drugs at the indicated final concentrations, alone or in combination. Cells are then isolated for processing at various times following drug exposure. The 96 well plate is centrifuged / cyto-spun to associate dead cells (for live-dead assays) with the base of each well. For live dead assays, after centrifugation, the media is removed and cells treated with live-dead reagent (Thermo Fisher Scientific, Waltham MA) and after 10 min this is removed and the cells in each well are visualized in the Hermes instrument at 10X magnification. Green cells = viable; yellow/red cells = dying/dead. The numbers of viable and dead cells were counted manually from three images taken from each well combined with data from another two wells of separately treated cells (i.e. the data is the mean cell dead from 9 data points from three separate exposures). For immuno-fluorescence studies, after centrifugation, the media is removed and cells are fixed in place and permeabilized using ice cold PBS containing 0.4% paraformaldehyde and 0.5% Triton X-100. After 30 min the cells are washed three times with ice cold PBS and cells are pre-blocked with rat serum for 3h. Cells are then incubated with a primary antibody to detect the expression / phosphorylation of a protein (usually at 1:100 dilution from a commercial vendor) overnight at 37°C. Cells are washed three times with PBS followed by application of the secondary antibody containing an associated fluorescent red or green chemical tag. After 3h of incubation the antibody is removed and the cells washed again. The cells are visualized at either 10X or 60X in the Hermes machine for imaging assessments. All immunofluorescent images for each individual protein / phospho-protein are taken using the identical machine settings so that the levels of signal in each image can be directly compared to the level of signal in the cells treated with drugs. Similarly, for presentation, the enhancement of image brightness/contrast using PhotoShop CS6 is simultaneously performed for each individual set of protein/phospho-protein to permit direct comparison of the image intensity between treatments. All immunofluorescent images were initially visualized at 75 dpi using an Odyssey infrared imager (Li-Cor, Lincoln, NE), then processed at 9999 dpi using Adobe Photoshop CS6. For presentation, immunoblots were digitally assessed using the provided Odyssey imager software. Images have their color removed and labeled figures generated in Microsoft PowerPoint.

#### Assessment of autophagy

Cells were transfected with a plasmid to express a green fluorescent protein (GFP) and red fluorescent protein (RFP) tagged form of LC3 (ATG8). For analysis of cells transfected with the GFP-RFP-LC3 construct, the GFP/RFP-positive vesicularized cells were examined under the ×40 objective of a Zeiss Axiovert fluorescent microscope.

### Animal studies

#### Neratinib/valproate

Studies were performed per USDA regulations under VCU IACUC protocol AD20008. 4T1 mouse TNBC cells (1 × 10^4^) were implanted into the rear flanks of female BALB/c mice and tumors permitted to form for 6 days until the mean tumor volume was ~25 mm^3^ [25]. Animals were then segregated into groups with near identical mean volumes and the animals then treated for three days with the indicated therapeutic agents: vehicle control (cremophore); neratinib 15 mg/kg (QD Days 1, 2, 3); sodium valproate 50 mg/kg (BID Days 1, 2, 3) or in combination. Two days after cessation of drug exposure animals are injected IP with: a control IgG (100 μg); an anti-PD-1 IgG (100 μg); or an anti-CTLA4 IgG (100 μg), as indicated. Tumor volumes were measured prior to drug administration and every three days after the initiation of therapeutic interventions. (n = 10 mice per group +/-SEM). Before, during and after drug treatment tumors are calipered as indicated in the Figure and tumor volume was assessed up to 20-40 days later. Animals were humanely sacrificed and the tumor and blood removed for further studies.

#### Pemetrexed/sorafenib/neratinib

Studies were performed per USDA regulations under VCU IACUC protocol AD20008. BT474 cells (2 × 10^6^) were implanted into the 4^th^ mammary fat pad of athymic mice. Lewis Lung Carcinoma cells (0.5 × 10^6^) were implanted into the rear flank of C57 black mice. Tumors permitted to form until the mean tumor volume was ~25 mm^3^. Animals were then segregated into groups with near identical mean volumes and the animals then treated for three days with the indicated therapeutic agents: vehicle control (cremophore); neratinib 15 mg/kg (QD Days 1, 2, 3); [sorafenib 20 mg/kg (BID Days 1, 2, 3) + pemetrexed 50 mg/kg (QD Day 1)] or the three drugs in combination. Tumor volumes were measured prior to drug administration and every three days after the initiation of therapeutic interventions. (n = 10 mice per group +/-SEM). Before, during and after drug treatment tumors are calipered as indicated in the Figure and tumor volume was assessed up to 20-40 days later. For BT474 cells when the volume of the tumor reached >500 mm^3^, animals were humanely sacrificed and the tumor and blood removed for further studies. For LLC cells, we discovered that when tumors grew beyond ~300 mm^3^ they became ulcerated and this necessitated the humane sacrifice of the animals.

#### Data analysis

Comparison of the effects of various treatments (performed in triplicate three times) was using one-way analysis of variance and a two tailed Student's t-test. Statistical examination of *in vivo* animal survival data utilized both a two tailed Student's t-test and log rank statistical analyses between the different treatment groups. Differences with a p-value of < 0.05 were considered statistically significant. Experiments shown are the means of multiple individual points from multiple experiments (± SEM).

## SUPPLEMENTARY MATERIALS FIGURES



## Abbreviations

ERK: extracellular regulated kinase
PI3K: phosphatidyl inositol 3 kinase
ca: constitutively active
dn: dominant negative
ER: endoplasmic reticulum
mTOR: mammalian target of rapamycin
JAK: Janus Kinase
STAT: Signal Transducers and Activators of Transcription
MAPK: mitogen activated protein kinase
PTEN: phosphatase and tensin homologue on chromosome ten
ROS: reactive oxygen species
CMV: empty vector plasmid or virus
si: small interfering
SCR: scrambled
IP: immunoprecipitation
VEH: vehicle
PTX: pemetrexed
SIL: sildenafil
SOR: sorafenib
HDAC: histone deacetylase
