# Correction: HDAC inhibitors enhance neratinib activity and when combined enhance the actions of an anti-PD-1 immunomodulatory antibody *in vivo*

**DOI:** 10.18632/oncotarget.27162

**Published:** 2019-08-20

**Authors:** Laurence Booth, Jane L. Roberts, Andrew Poklepovic, Francesca Avogadri-Connors, Richard E. Cutler, Alshad S. Lalani, Paul Dent

**Affiliations:** ^1^ Department of Biochemistry and Molecular Biology, Virginia Commonwealth University, Richmond, VA 23298, USA; ^2^ Department of Medicine, Virginia Commonwealth University, Richmond, VA 23298, USA; ^3^ Puma Biotechnology Inc., Los Angeles, CA 90024, USA

**This article has been corrected:** Due to errors in image preparation, the separate red/green images presented for vehicle control and valproate in Figure 6A were incorrect. In addition, in Figure 9, the graphical panel on the left has been clarified and an extra sentence and clauses have been added into the figure legend which completely and accurately describe how the in vivo studies were performed. Both corrected figures are shown below. The authors declare that these corrections do not change the results or conclusions of this paper.

Original article: Oncotarget. 2017; 8:90262–90277. 90262-90277 . https://doi.org/10.18632/oncotarget.21660

**Figure 6:**
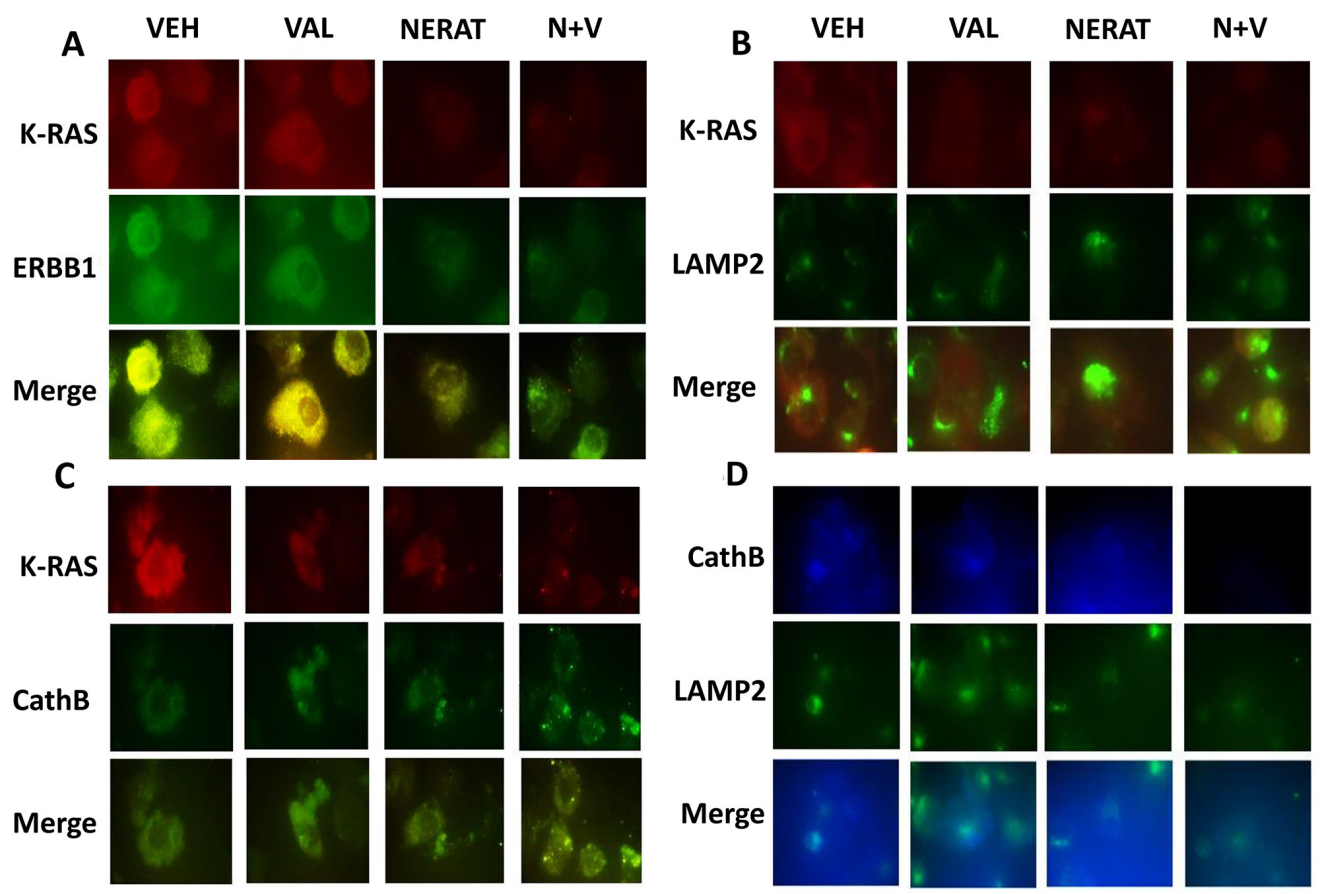
Figure 6: Neratinib promotes the co-localization of K-RAS with LAMP2 and cathepsin B, and the disassociation of K-RAS and ERBB1. (**A**) PANC-1 cells were treated with vehicle control, sodium valproate (250 μM), neratinib (0.5 μM) or the drugs combined for 6h. Cells were fixed in place and immunostaining performed to determine the co-localization of K-RAS with ERBB1 at 60X magnification. (**B**) PANC-1 cells were treated with vehicle control, sodium valproate (250 μM), neratinib (0.5 μM) or the drugs combined for 6h. Cells were fixed in place and immunostaining performed to determine the co-localization of K-RAS with LAMP2 at 60X magnification. (**C**) PANC-1 cells were treated with vehicle control, sodium valproate (250 μM), neratinib (0.5 μM) or the drugs combined for 6h. Cells were fixed in place and immunostaining performed to determine the co-localization of K-RAS with cathepsin B at 60X magnification. (**D**) PANC-1 cells were treated with vehicle control, sodium valproate (250 μM), neratinib (0.5 μM) or the drugs combined for 6h. Cells were fixed in place and immunostaining performed to determine the co-localization of LAMP2 and cathepsin B at 60X magnification.

**Figure 6:**
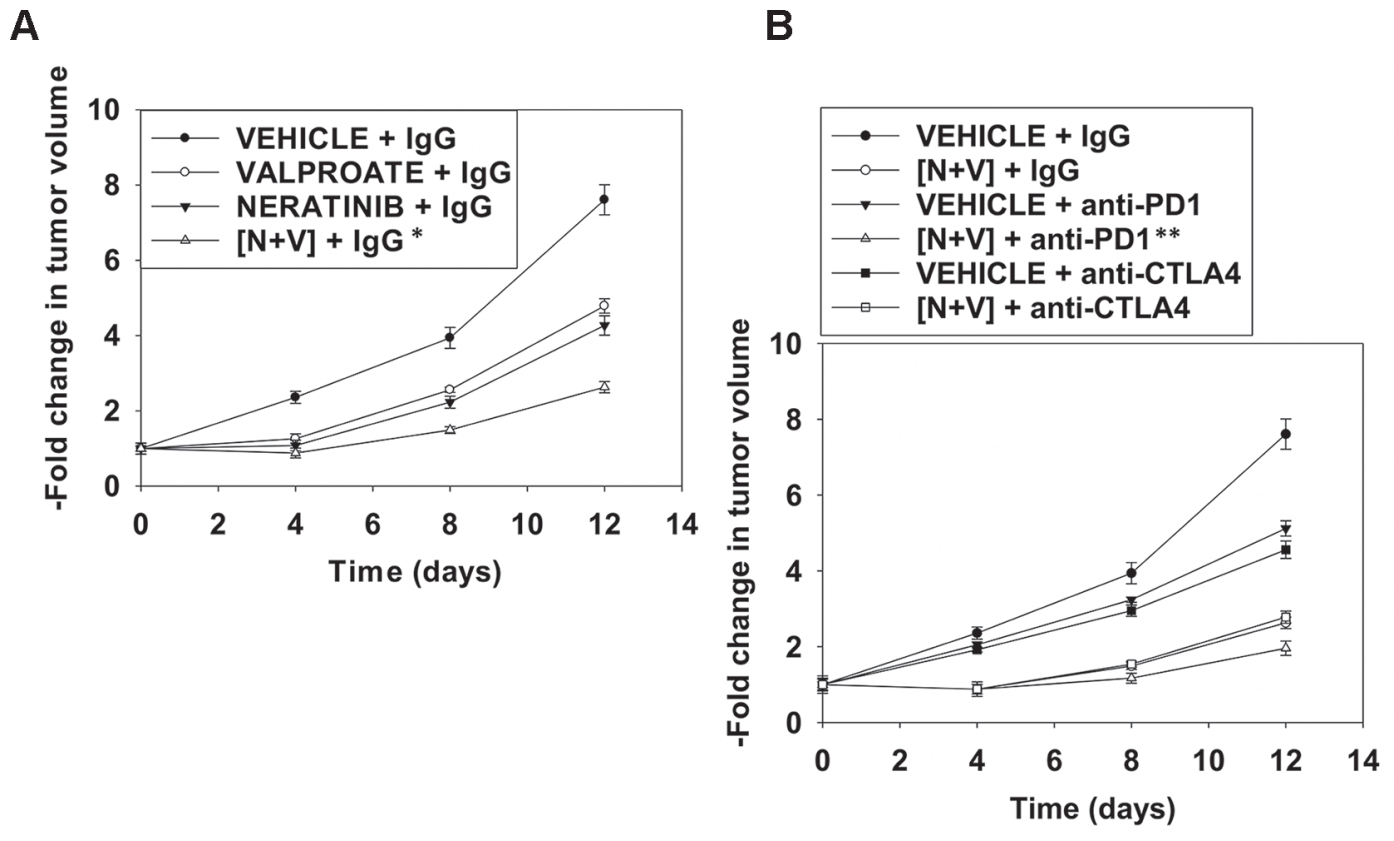
Figure 9: Neratinib and valproate interact to suppress tumor growth and to opsonize the surviving tumor cells to checkpoint immunotherapies. (**A**) and (**B**) BALB/c mice were implanted with 4T1 cells in the 4^th^ mammary fat pad and ~30 mm^3^ tumors permitted to form. Animals were then treated with vehicle control, neratinib (15 mg/kg QD), valproate (50 mg/kg BID) or the drugs in combination for 3 days. Two days after the cessation of drug exposure mice were injected IP with a control IgG (100 μg / mouse); an anti-PD-1 antibody (100 μg / mouse); or an anti-CTLA4 antibody (100 μg / mouse). Tumor volumes were measured prior to drug administration and every three days after the initiation of therapeutic interventions. (n = 10 mice per group +/-SEM). * p < 0.05 less than neratinib alone or valproate alone; ** p < 0.05 less than IgG + [neratinib + valproate].

