# Supplementary material for: HDAC inhibitors enhance neratinib activity and when combined enhance the actions of an anti-PD-1 immunomodulatory antibody in vivo
Source: Oncotarget. 2017 Oct 9;8(52):90262–77. doi: 10.18632/oncotarget.21660 (PMC5685747; doi:10.18632/oncotarget.21660)
Supplement: Supplementary file 1 [file oncotarget-08-90262-s001.pdf]

HDAC inhibitors enhance neratinib activity and when combined enhance the actions of an anti-PD-1 immunomodulatory antibody *in vivo*

SUPPLEMENTARY MATERIALS

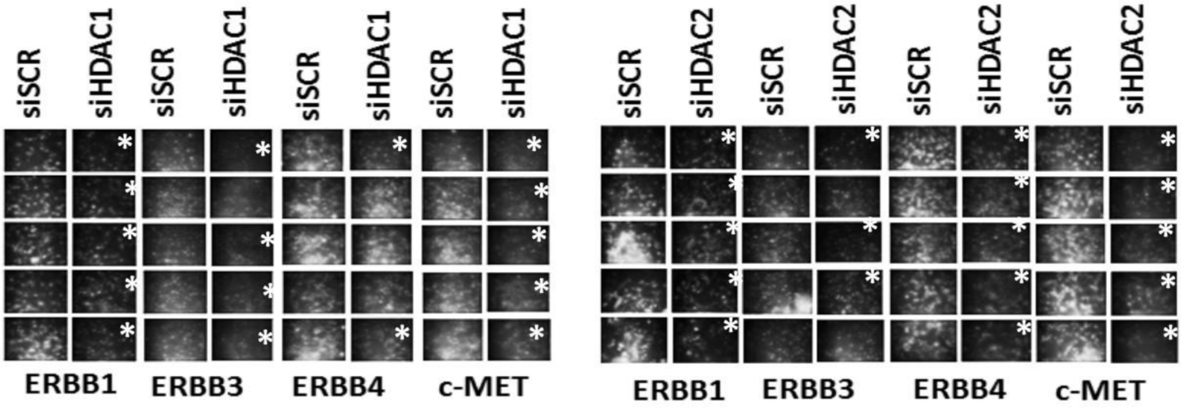

Supplementary Figure 1: Neratinib down-regulates the expression of histone deacetylase enzymes. Afatinib resistant H1975 clones were transfected with a scrambled siRNA or with siRNA molecules to knock down HDAC1 or to knock down HDAC2. Twenty-four h after transfection cells were fixed in place and immunostaining performed to determine the expression of ERBB1, ERBB3, ERBB4 and c-MET. (n = 3 +/-SEM) * p < 0.05 less staining intensity than corresponding siSCR value.

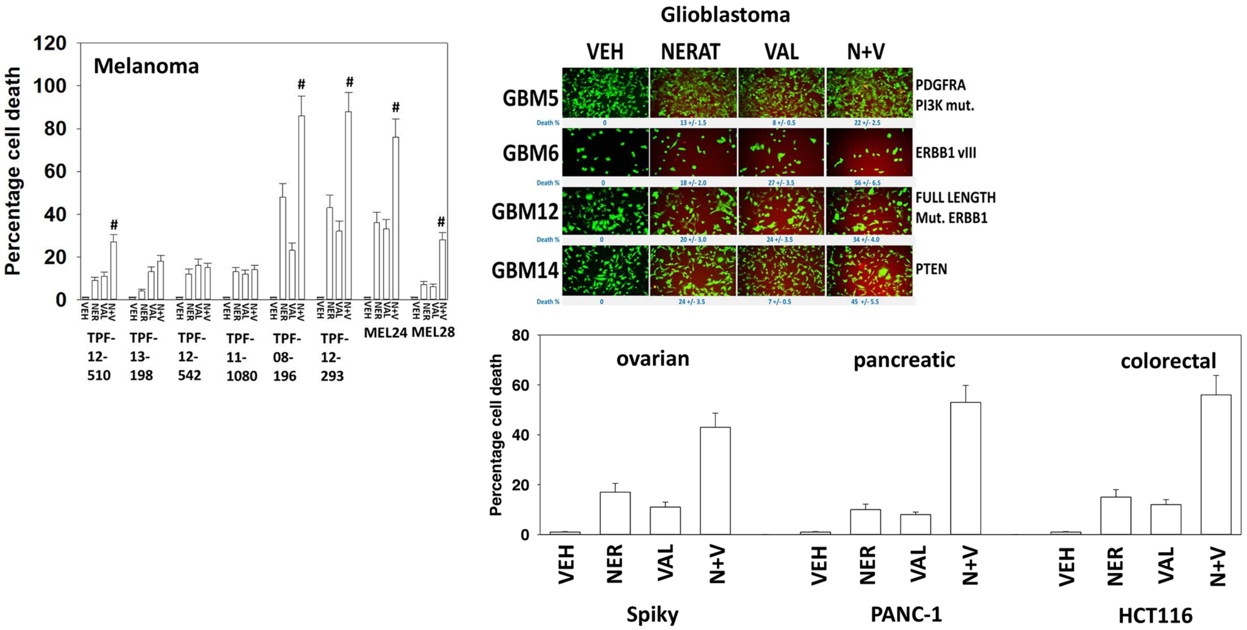

Supplementary Figure 2: Neratinib and sodium valproate interact to kill tumor cells expressing mutant B-RAF, mutant K-RAS, mutant N-RAS, expressing ERBB1 vIII and lacking PTEN. Tumor cells, as indicated in the Figure, were treated with vehicle control, neratinib (0.5 μM), valproate (250 μM) or the drugs in combination for 24h. Cell viability was then determined by live/dead assay. (n = 3 +/-SEM) # p < 0.05 greater than corresponding neratinib value. The melanoma cells all express a mutated active B-RAF. The ovarian cancer cells express a mutant N-RAS and the pancreatic and colorectal lines express a mutant K-RAS.

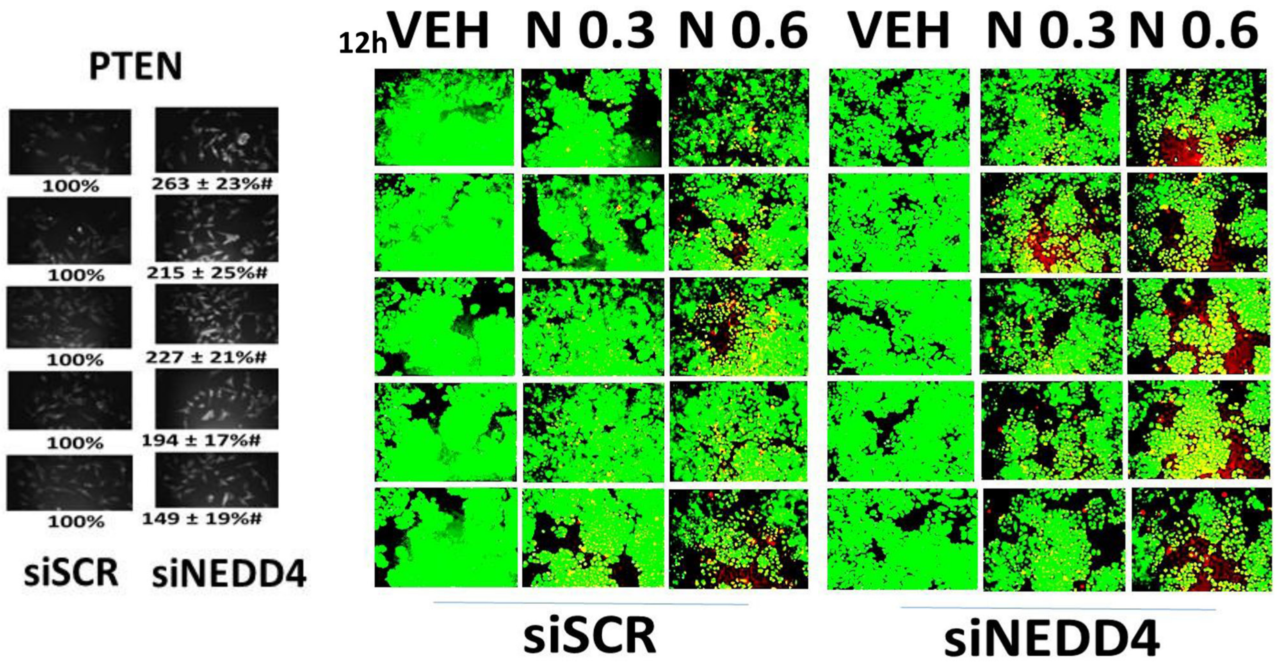

Supplementary Figure 3: Knock down of NEDD4 enhances neratinib lethality in afatinib resistant clones. Left: Afatinib resistant clones were transfected with a scrambled siRNA or an siRNA to knock down NEDD4. Twenty-four h after transfection, cells were fixed in place and immunostaining performed to determine the total expression of PTEN. (n = 3 +/-SEM) # $p < 0.05$ greater than corresponding siSCR value. Right: Afatinib resistant clones were transfected with a scrambled siRNA or an siRNA to knock down NEDD4. Twenty-four h after transfection, cells were treated with vehicle control, neratinib (0.3 µM) or neratinib (0.6 µM). (n = 3 +/-SEM) # $p < 0.05$ greater than corresponding neratinib value.

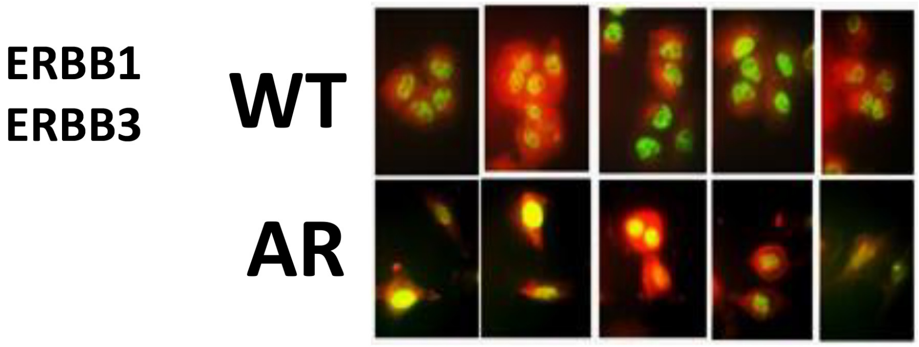

Supplementary Figure 4: ERBB1 and ERBB3 co-localize in afatinib resistant H1975 clones. Wild type parental and afatinib resistant H1975 clones, 24h after plating, were fixed in place. Immunostaining was performed to detect the expression of ERBB1 and of ERBB3 at 60X magnification. The images were over-layered and merged in Adobe Photoshop CS6.

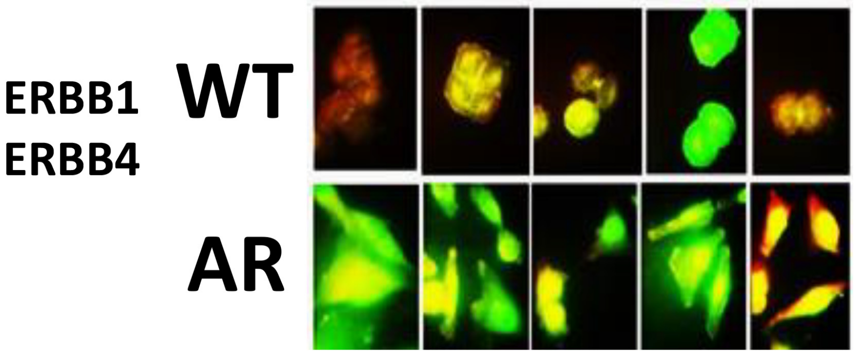

Supplementary Figure 5: ERBB1 and ERBB4 co-localize in wild type parental and in afatinib resistant H1975 clones. Wild type parental and afatinib resistant H1975 clones, 24h after plating, were fixed in place. Immunostaining was performed to detect the expression of ERBB1 and of ERBB4 at 60X magnification. The images were over-layered and merged in Adobe Photoshop CS6.

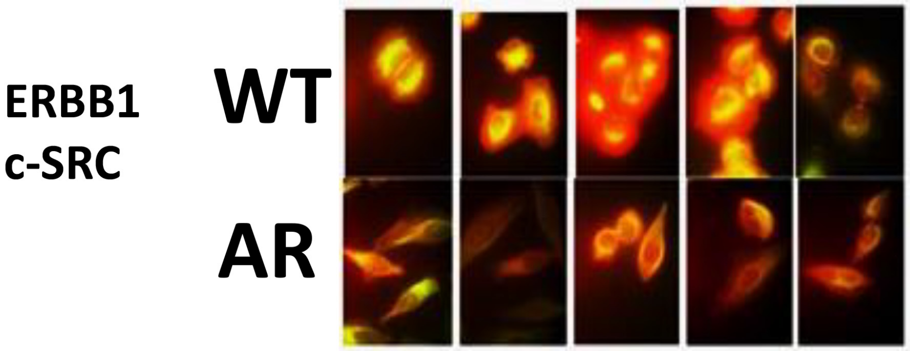

Supplementary Figure 6: ERBB1 and c-SRC co-localize in afatinib resistant H1975 clones. Wild type parental and afatinib resistant H1975 clones, 24h after plating, were fixed in place. Immunostaining was performed to detect the expression of ERBB1 and of c-SRC at 60X magnification. The images were over-layered and merged in Adobe Photoshop CS6.

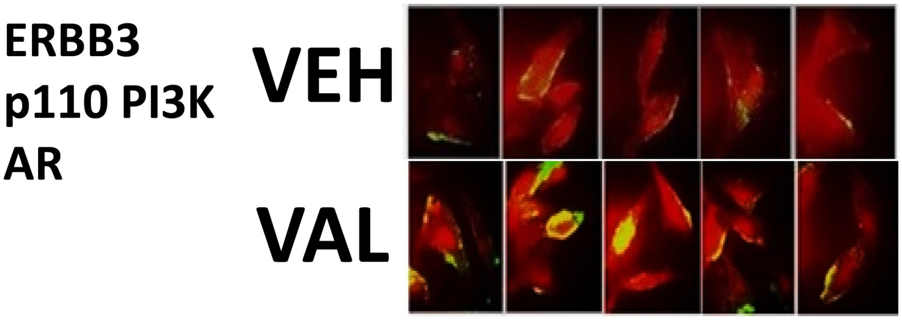

Supplementary Figure 7: Valproate promotes c-SRC co-localization with ERBB3 in afatinib resistant clones. Afatinib resistant H1975 clones, 24h after plating, were treated with vehicle control or with sodium valproate (250 μM), and after 6h, fixed in place. Immunostaining was performed to detect the expression of ERBB3 and of PI3K p110α/β at 60X magnification. The images were over-layered and merged in Adobe Photoshop CS6.

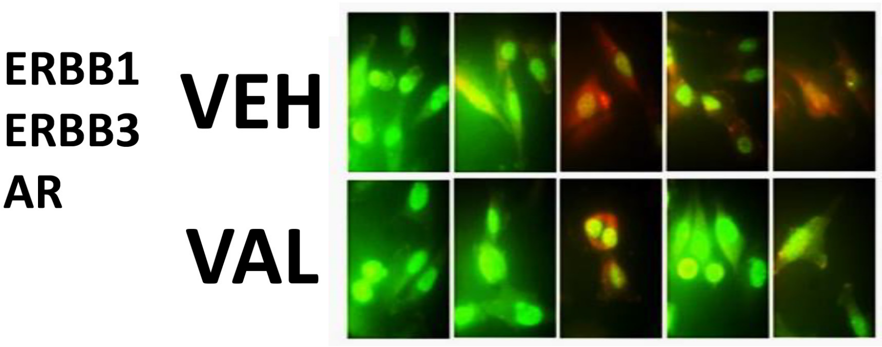

Supplementary Figure 8: Valproate promotes ERBB1 co-localization with ERBB3 in afatinib resistant clones. Afatinib resistant H1975 clones, 24h after plating, were treated with vehicle control or with sodium valproate (250 μM), and after 6h, fixed in place. Immunostaining was performed to detect the expression of ERBB3 and of ERBB1 at 60X magnification. The images were over-layered and merged in Adobe Photoshop CS6.

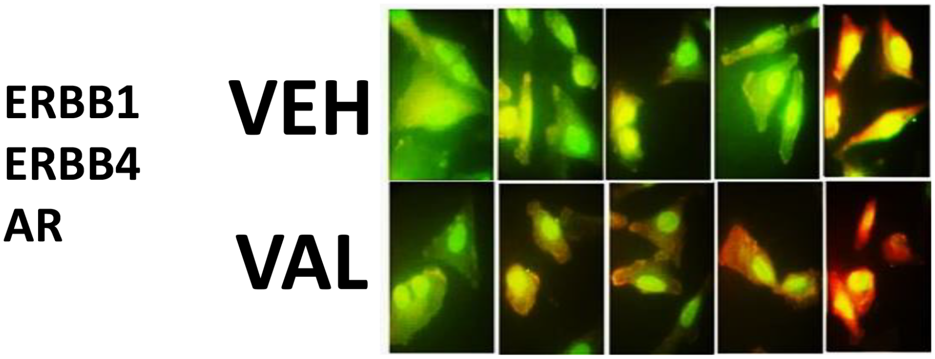

Supplementary Figure 9: Valproate reduces ERBB1 co-localization with ERBB4 in afatinib resistant clones. Afatinib resistant H1975 clones, 24h after plating, were treated with vehicle control or with sodium valproate (250 μM), and after 6h, fixed in place. Immunostaining was performed to detect the expression of ERBB1 and of ERBB4 at 60X magnification. The images were over-layered and merged in Adobe Photoshop CS6.

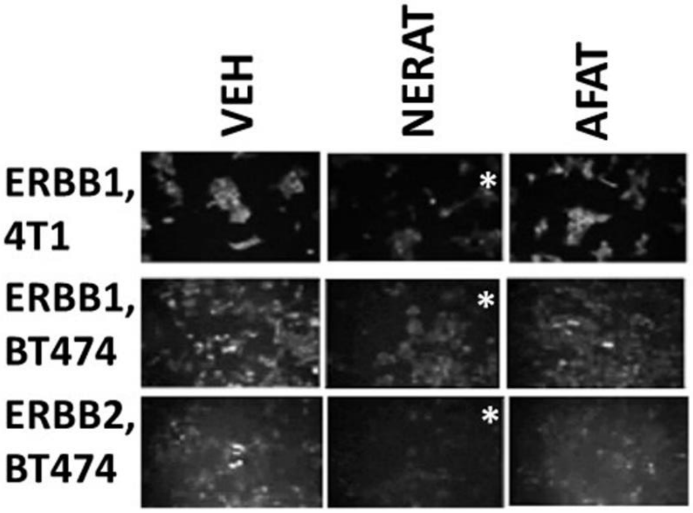

Supplementary Figure 10: Neratinib, but not afatinib, reduces the expression of ERBB1 and ERBB2 in mammary carcinoma cells. BT474 and 4T1 mammary carcinoma cells were treated with vehicle control, neratinib (0.5 μM) or afatinib (0.5 μM). Cells were fixed in place 6h later and immunostaining performed to determine the expression of ERBB1 and ERBB2 (n = 3 +/-SEM) * $p < 0.05$ less than corresponding vehicle control value.

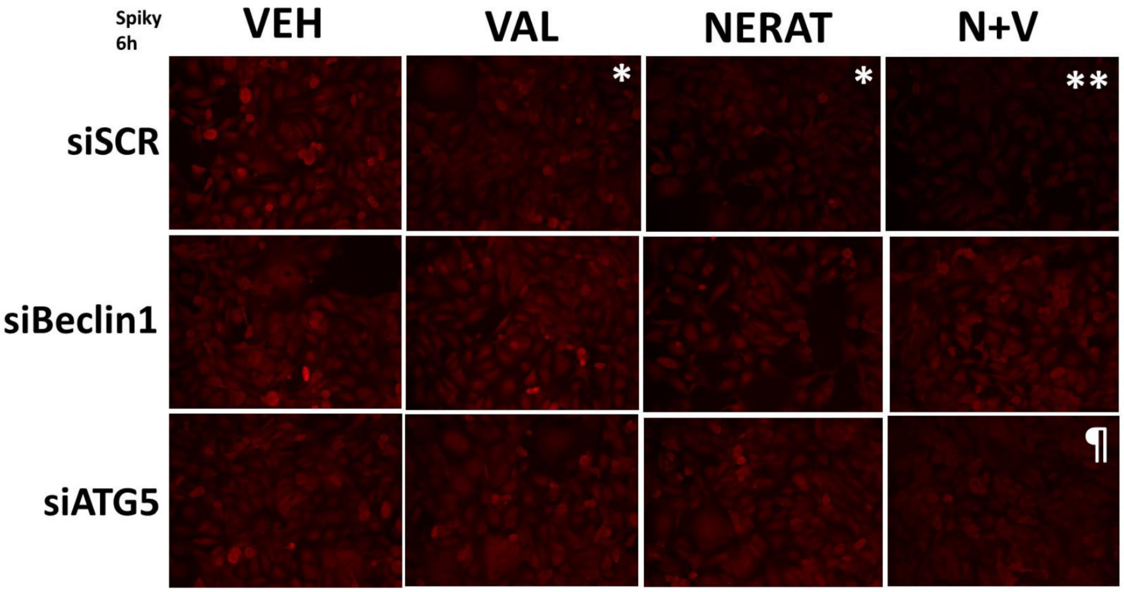

Supplementary Figure 11: Neratinib, and to a greater extent [neratinib + valproate], reduce the expression of N-RAS in ovarian cancer cells expressing a mutated N-ARS. Spiky ovarian tumor cells were treated with vehicle control, neratinib (0.5 µM), sodium valproate (250 µM) or the drugs in combination for 6h. Cells were fixed in place and immunofluorescent staining performed to detect the total cellular expression of N-RAS (n = 3 +/-SEM) * $p < 0.05$ less than vehicle control value; ** $p < 0.05$ less than neratinib value; ¶ $p < 0.05$ greater than corresponding treatment in siSCR and less than corresponding value in siBeclin1 cells.

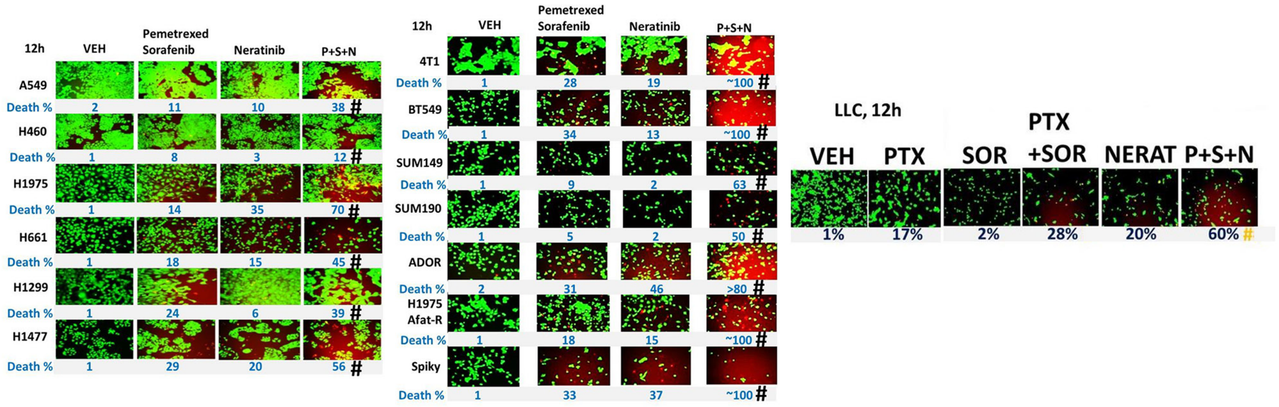

Supplementary Figure 12: Neratinib enhances [pemetrexed + sorafenib] lethality. Tumor cells were treated with vehicle control, [pemetrexed (0.5 µM) + sorafenib (2.0 µM)], neratinib (0.5 µM) or the drugs in combination for 12h. Cells were then treated with live/dead reagent and cell viability determined (n = 3 +/-SEM) # $p < 0.05$ greater than [pemetrexed + sorafenib] value.

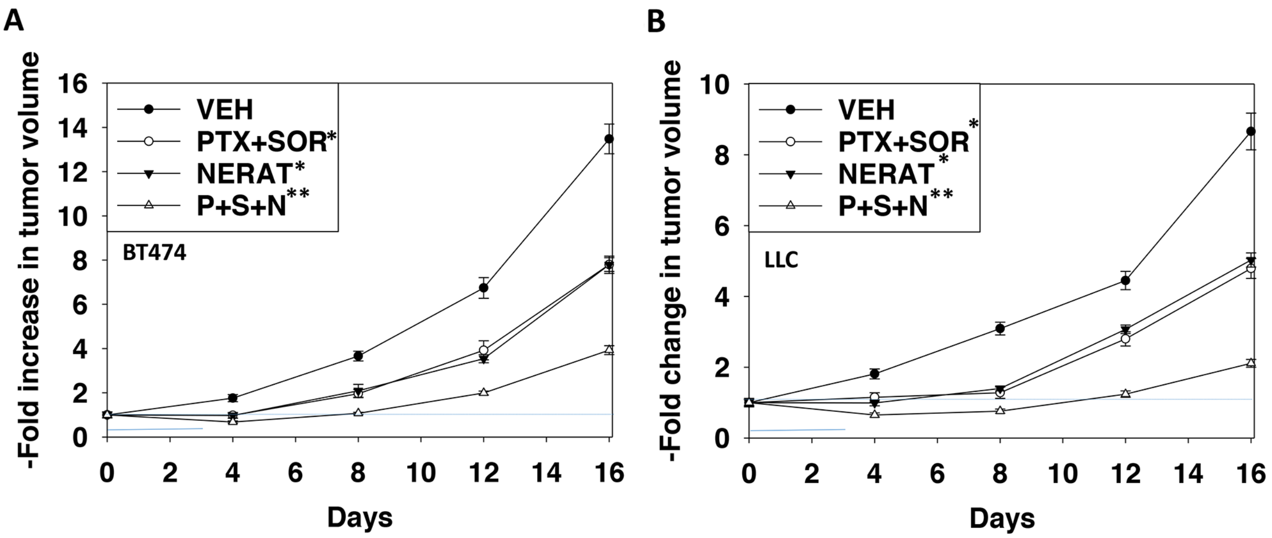

Supplementary Figure 13: Neratinib enhances [pemetrexed + sorafenib] lethality *in vivo*. **(A)** BT474 tumors were formed in the 4th mammary fat pad of athymic mice. As described in the Methods, animals were treated with vehicle control, [pemetrexed + sorafenib], neratinib, or the three drugs together for 3 days. Tumors were calipered every 4 days (n = 10 per group +/-SEM) * $p < 0.05$ less growth than vehicle control; ** $p < 0.05$ less growth than [pemetrexed + sorafenib]. **(B)** Lewis Lung Carcinoma tumors were formed in the rear flank of C57 black mice. As described in the Methods, animals were treated with vehicle control, [pemetrexed + sorafenib], neratinib, or the three drugs together for 3 days. Tumors were calipered every 4 days (n = 10 per group +/-SEM) * $p < 0.05$ less growth than vehicle control; ** $p < 0.05$ less growth than [pemetrexed + sorafenib].

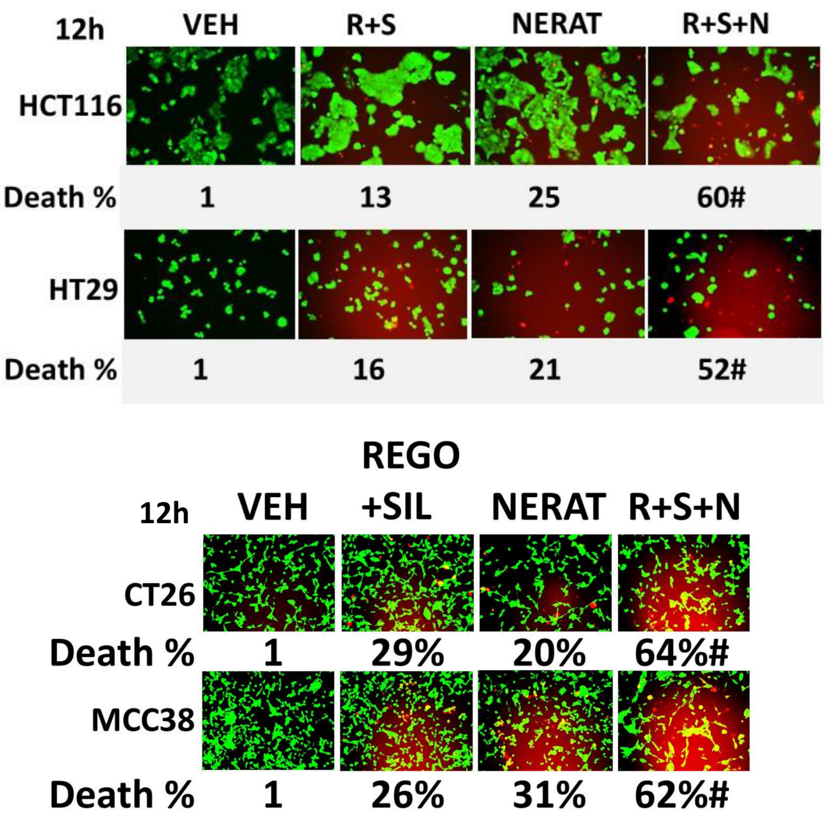

Supplementary Figure 14: Neratinib enhances [regorafenib + sildenafil] lethality. Colon cancer cells were treated with vehicle control, [regorafenib (0.5 µM) + sildenafil (2.0 µM)], neratinib (0.5 µM) or the three drugs combined for 12h. Cell viability was determined by live/dead assay (n = 3 +/-SEM) # $p < 0.05$ greater than [regorafenib + sildenafil] value.

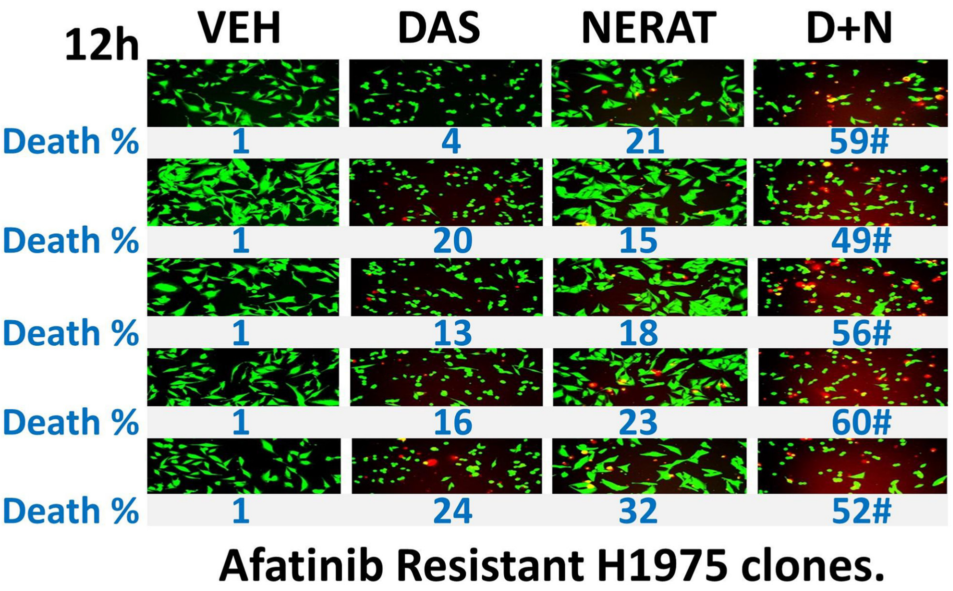

Supplementary Figure 15: Neratinib interacts with the c-SRC inhibitor dasatinib to kill afatinib resistant NSCLC cells. Afatinib H1975 cells were treated with vehicle control, neratinib (0.5 µM), dasatinib (0.5 µM) or the drug combined for 12h. Cell viability was determined by live/dead assay (n = 3 +/-SEM) # $p < 0.05$ greater than neratinib alone value.

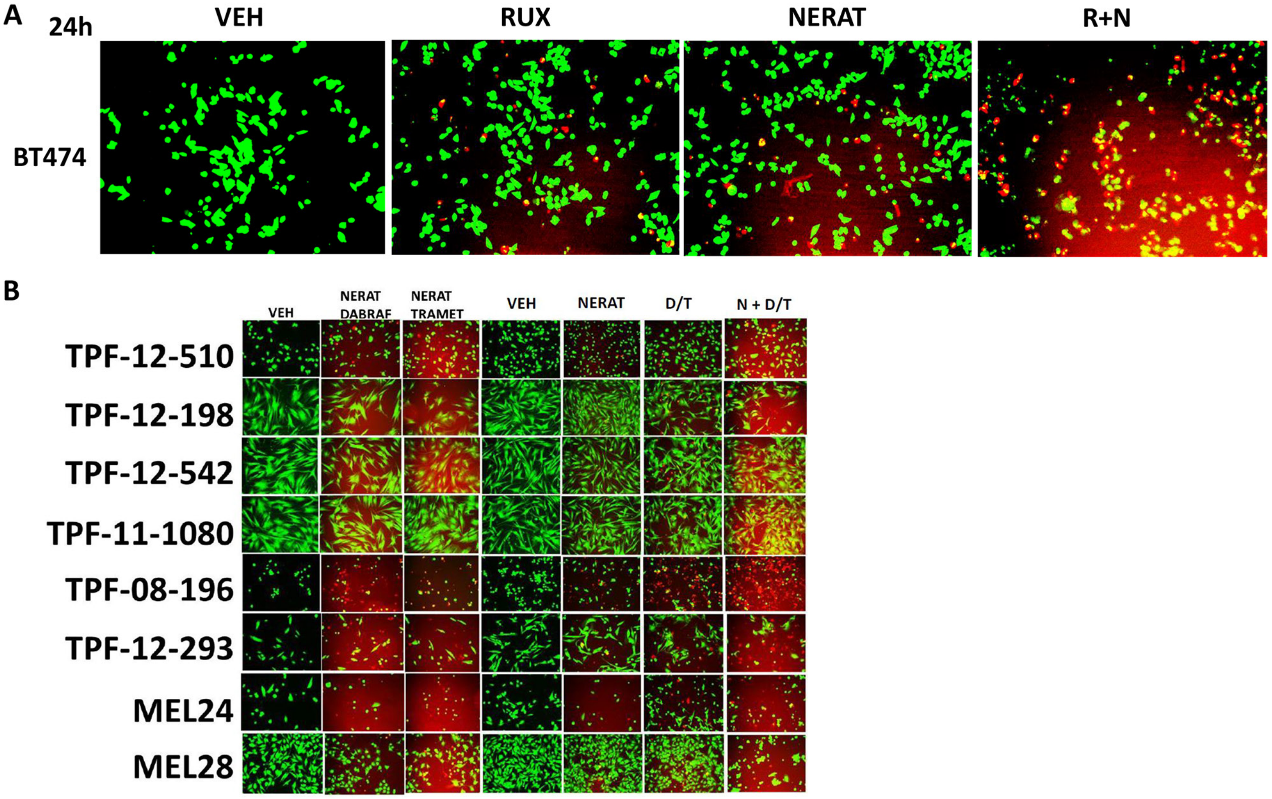

Supplementary Figure 16: Neratinib facilitates cell killing by ruxolitinib, dabrafenib and trametinib. **(A)** BT474 mammary carcinoma cells were treated with vehicle control, neratinib (0.5 mM), ruxolitinib (0.5 mM) or the drugs in combination for 24h. Cell viability was determined by live/dead assay where green staining cells are viable and yellow/red cells are dying/dead. **(B)** Melanoma cells expressing a mutated active B-RAF protein were treated with vehicle control, neratinib (0.5 µM), dabrafenib (0.5 µM), trametinib (0.5 µM) or the drugs in combination as indicated for 24h. Cell viability was determined by live/dead assay where green staining cells are viable and yellow/red cells are dying/dead.

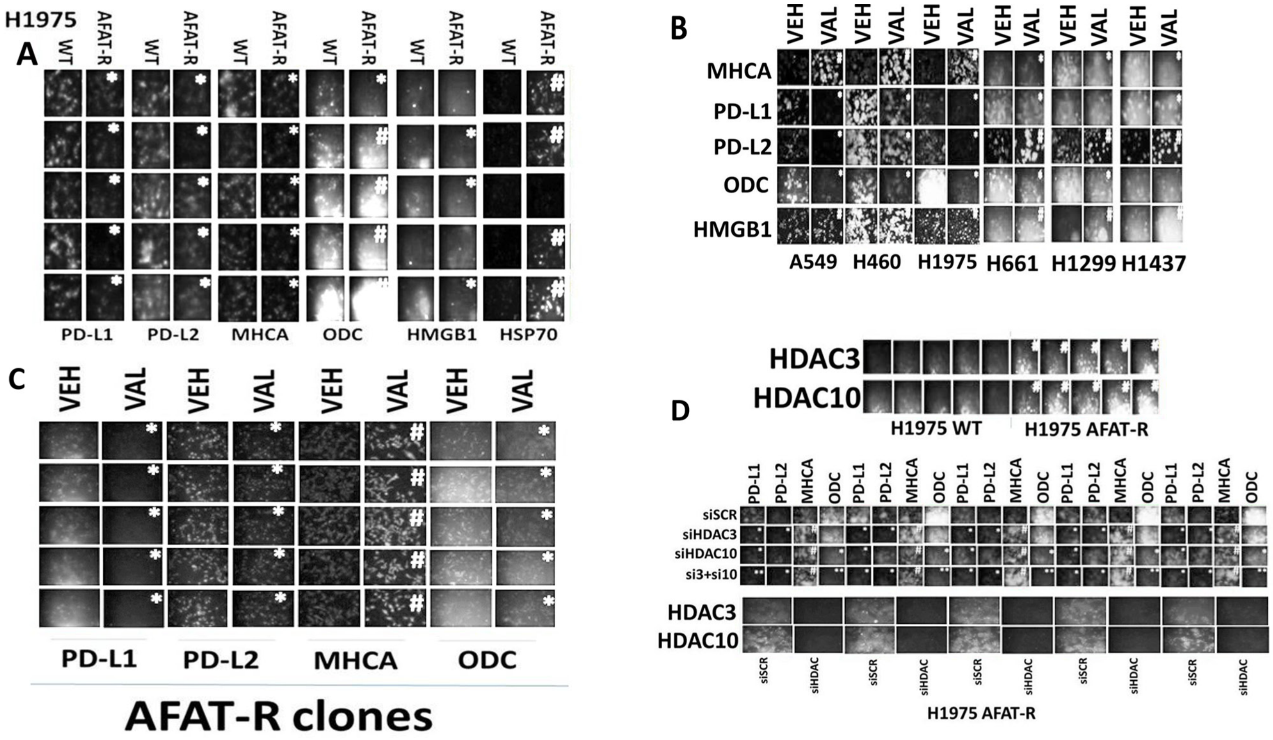

Supplementary Figure 17: Sodium valproate modulates the expression of immunotherapy biomarkers in part via inhibition of HDAC3 and HDAC10. (A) Wild type parental and afatinib resistant cells, 24h after plating cells were fixed in place and immunostaining performed to determine the expression of PD-L1, PD-L2, MHCA, ODC, HMGB1 and HSP70. (n = 3 +/-SEM) * $p < 0.05$ less intensity of staining compared to wild type parental cells; # $p < 0.05$ greater intensity of staining compared to wild type parental cells. **(B)** NSCLC cells were treated for 6h with vehicle control or with sodium valproate (250 μM). Cells were then fixed in place and immunostaining performed to determine the expression levels of PD-L1, PD-L2, MHCA, ODC, HMGB1 and HSP70. (n = 3 +/-SEM) * $p < 0.05$ less intensity of staining compared to vehicle control cells; # $p < 0.05$ greater intensity of staining compared to wild type parental cells. **(C)** Afatinib resistant clones were treated for 6h with vehicle control or with sodium valproate (250 μM). Cells were then fixed in place and immunostaining performed to determine the expression levels of PD-L1, PD-L2, MHCA, ODC, HMGB1 and HSP70. (n = 3 +/-SEM) * $p < 0.05$ less intensity of staining compared to vehicle control cells; # $p < 0.05$ greater intensity of staining compared to wild type parental cells. **(D)** Afatinib resistant clones were transfected with a scrambled siRNA control or with siRNA molecules to knock down the expression of HDAC3 or HDAC10, or HDAC3 and HDAC10 together. Twenty-four h after transfection, cells were fixed in place to determine the expression levels of PD-L1, PD-L2, MHCA, ODC, HMGB1 and HSP70. (n = 3 +/-SEM) * $p < 0.05$ less intensity of staining compared to siSCR cells; ** $p < 0.01$ less intensity of staining compared to siSCR cells; # $p < 0.05$ greater intensity of staining compared to siSCR cells.

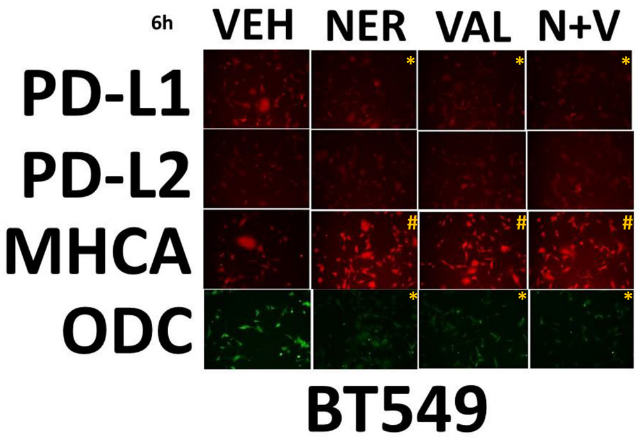

Supplementary Figure 18: Neratinib and valproate regulate the expression of immunotherapy biomarkers in human TNBC cells. BT549 cells were treated with vehicle control, neratinib (0.5 μM), sodium valproate (250 μM) or the drugs in combination for 6h. Cells were fixed in place and immunostaining performed to determine the expression of PD-L1, PD-L2, MHCA and ornithine decarboxylase (ODC). (n = 3 +/-SEM) * p < 0.05 less staining intensity than vehicle control; # p < 0.05 greater staining intensity than vehicle control.

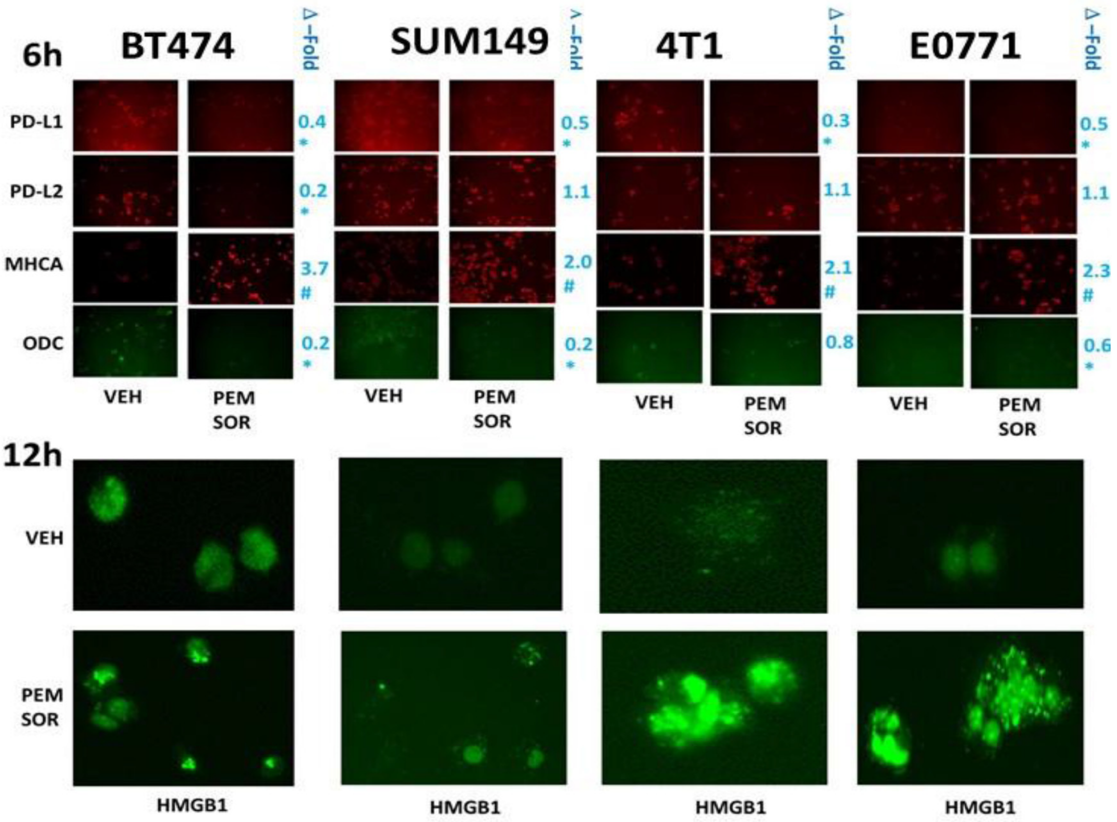

Supplementary Figure 19: [Pemetrexed + sorafenib] regulates the expression of immunotherapy biomarkers. Mammary carcinoma cells were treated with vehicle control or with [pemetrexed (0.5 μM) + sorafenib (2.0 μM)] for 6h or 12h. Cells were fixed in place and immunostaining performed to determine the expression of PD-L1, PD-L2, MHCA and ornithine decarboxylase (ODC). (n = 3 +/-SEM) * p < 0.05 less staining intensity than vehicle control; # p < 0.05 greater staining intensity than vehicle control.

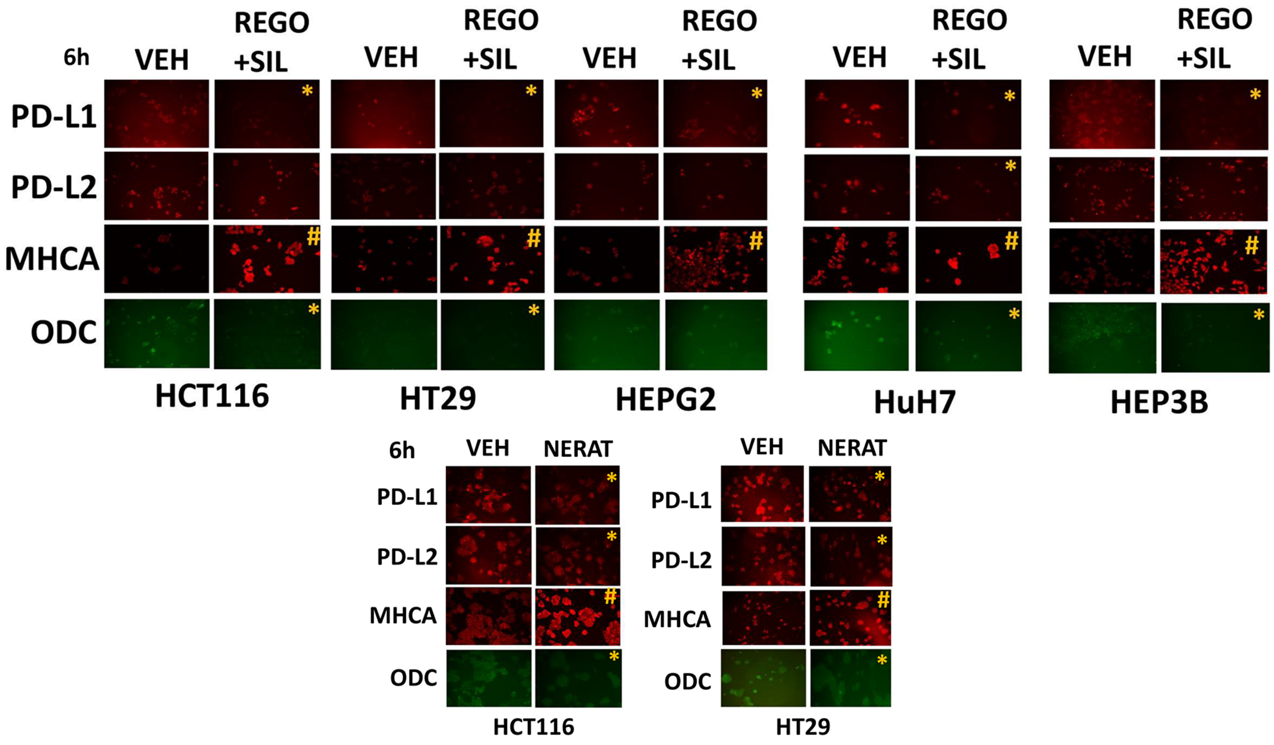

Supplementary Figure 20: [Regorafenib + sildenafil] regulates the expression of immunotherapy biomarkers. Liver and colorectal carcinoma cells were treated with vehicle control or with [regorafenib (0.5 μM) + sildenafil (2.0 μM)] for 6h. Cells were fixed in place and immunostaining performed to determine the expression of PD-L1, PD-L2, MHCA and ornithine decarboxylase (ODC). (n = 3 +/-SEM) * $p < 0.05$ less staining intensity than vehicle control; # $p < 0.05$ greater staining intensity than vehicle control.

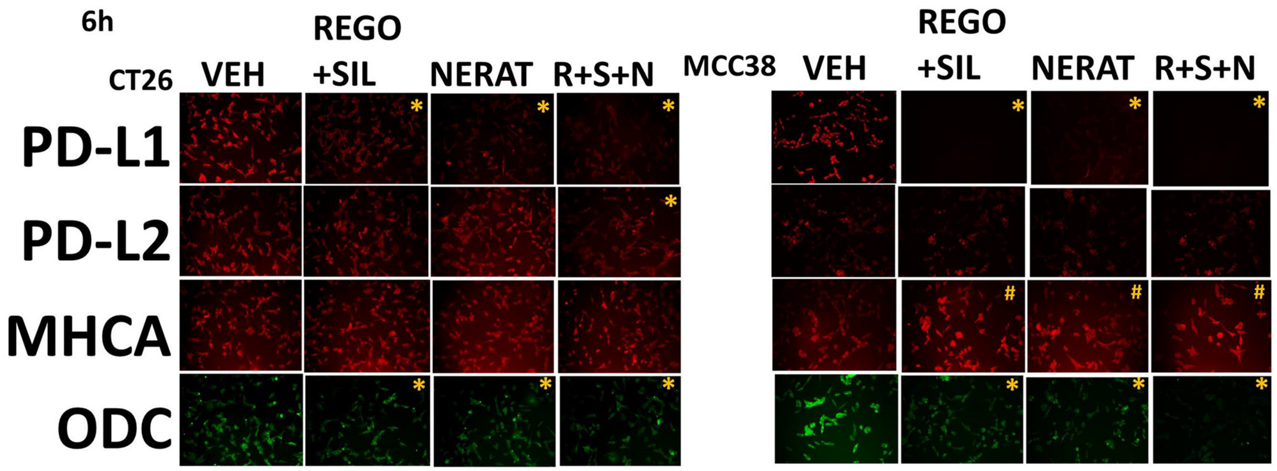

Supplementary Figure 21: [Regorafenib + sildenafil] and neratinib regulates the expression of immunotherapy biomarkers. Mouse colorectal carcinoma cells were treated with vehicle control or with [regorafenib (0.5 μM) + sildenafil (2.0 μM)], neratinib (0.5 μM) or the drugs in combination for 6h. Cells were fixed in place and immunostaining performed to determine the expression of PD-L1, PD-L2, MHCA and ornithine decarboxylase (ODC). (n = 3 +/-SEM) * $p < 0.05$ less staining intensity than vehicle control; # $p < 0.05$ greater staining intensity than vehicle control.

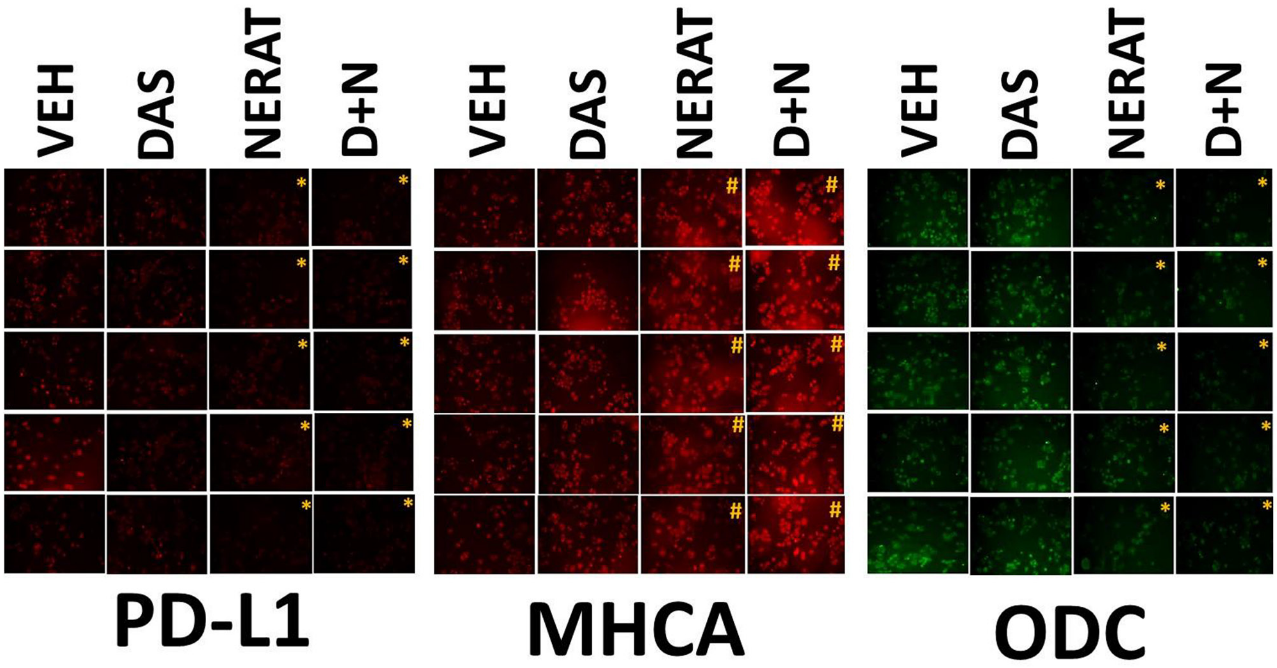

Supplementary Figure 22: [Neratinib + dasatinib] regulates the expression of immunotherapy biomarkers. Afatinib resistant H1975 clones were treated with vehicle control or with dasatinib (0.5 µM), neratinib (0.5 µM) or the drugs in combination for 6h. Cells were fixed in place and immunostaining performed to determine the expression of PD-L1, PD-L2, MHCA and ornithine decarboxylase (ODC). (n = 3 +/-SEM) * $p < 0.05$ less staining intensity than vehicle control; # $p < 0.05$ greater staining intensity than vehicle control.

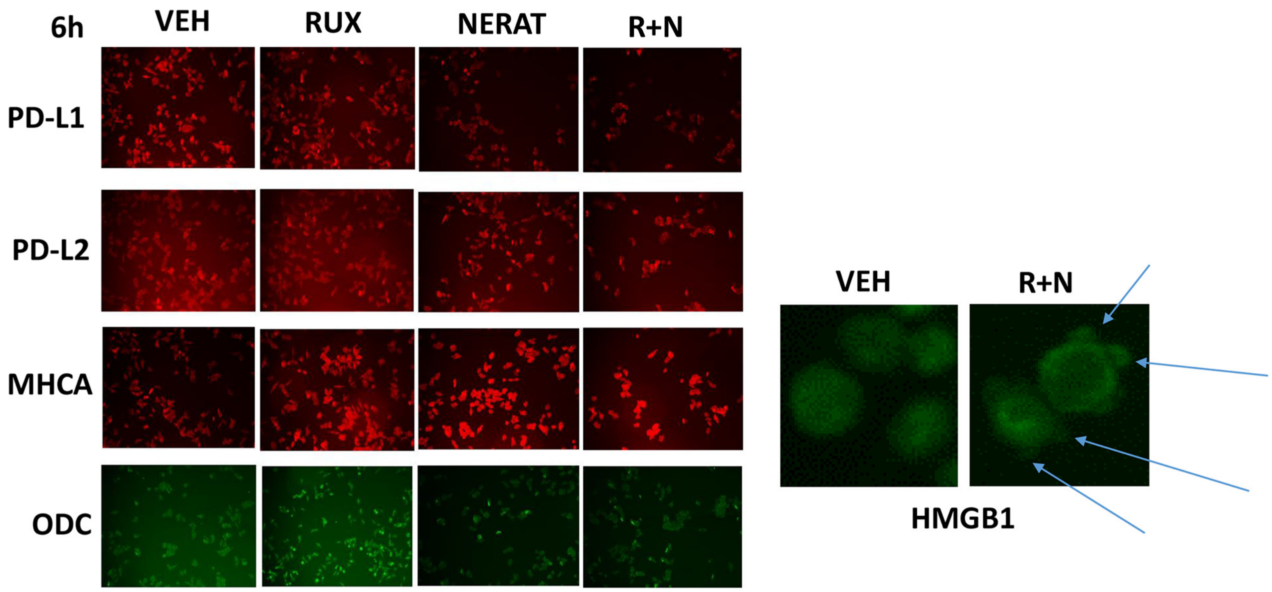

Supplementary Figure 23: [Ruxolitinib + neratinib] interact to regulate the expression of immunotherapy biomarkers and cause the extracellular release of HMGB1. BT549 cells were treated with vehicle control or with ruxolitinib (0.5 µM), neratinib (0.5 µM) or the drugs in combination for 6h. Cells were fixed in place and immunostaining performed to determine the expression of PD-L1, PD-L2, MHCA and ornithine decarboxylase (ODC). (n = 3 +/-SEM) * $p < 0.05$ less staining intensity than vehicle control; # $p < 0.05$ greater staining intensity than vehicle control. The inset panel shows the drug combination causing extracellular release of HMGB1.

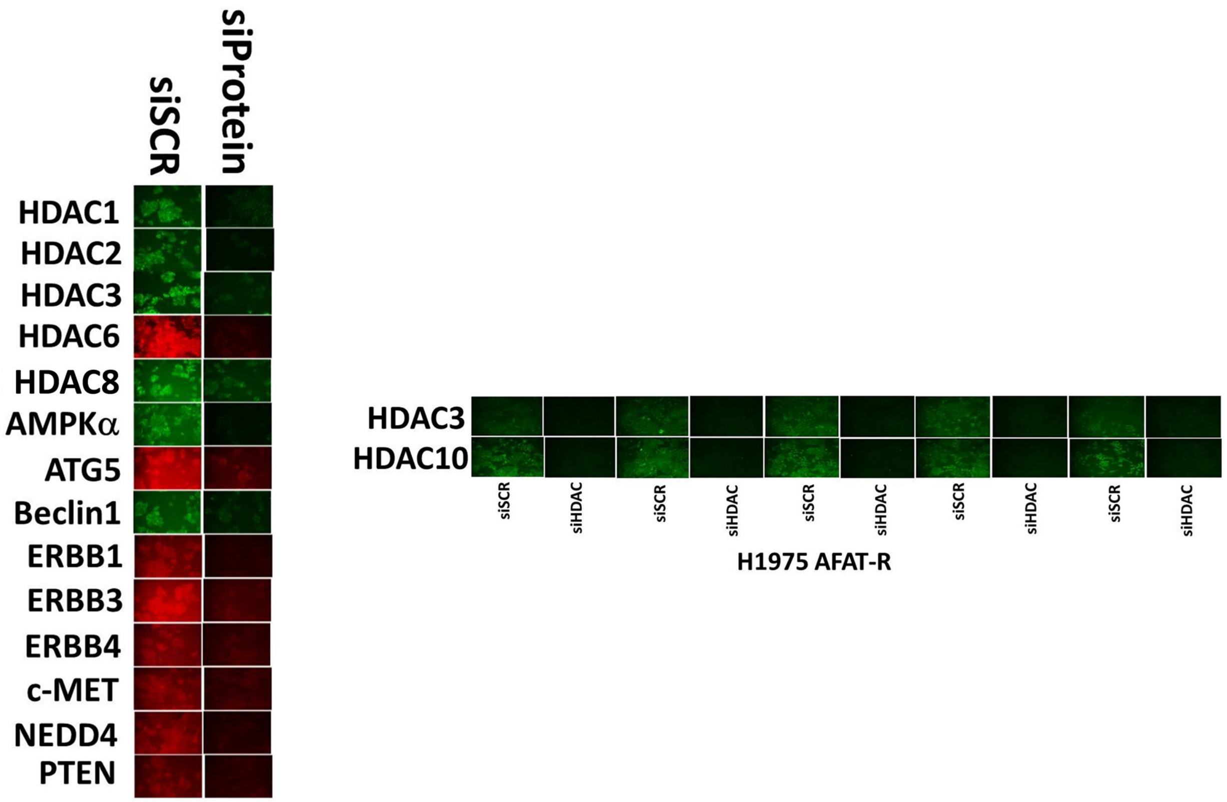

Supplementary Figure 24: Control immunofluorescence images showing the knock down of various proteins. Cells were transfected with a scrambled siRNA or an siRNA to knock down the indicated protein. Cells were fixed in place 24h after transfection and immunostaining performed. The extent of knock down varied between 60% and 90%.
